# MixingDTA: improved drug–target affinity prediction by extending mixup with guilt-by-association

**DOI:** 10.1093/bioinformatics/btaf238

**Published:** 2025-07-15

**Authors:** Youngoh Kim, Dongmin Bang, Bonil Koo, Jungseob Yi, Changyun Cho, Jeonguk Choi, Sun Kim

**Affiliations:** Bio-MAX Institute, Seoul National University, Seoul, 08826, Republic of Korea; Interdisciplinary Program in Bioinformatics, Seoul National University, Seoul, 08826, Republic of Korea; AIGENDRUG Co., Ltd., Seoul, 08758, Republic of Korea; Interdisciplinary Program in Bioinformatics, Seoul National University, Seoul, 08826, Republic of Korea; AIGENDRUG Co., Ltd., Seoul, 08758, Republic of Korea; Interdisciplinary Program in Artificial Intelligence, Seoul National University, Seoul, 08826, Republic of Korea; Interdisciplinary Program in Bioinformatics, Seoul National University, Seoul, 08826, Republic of Korea; AIGENDRUG Co., Ltd., Seoul, 08758, Republic of Korea; Interdisciplinary Program in Artificial Intelligence, Seoul National University, Seoul, 08826, Republic of Korea; Interdisciplinary Program in Bioinformatics, Seoul National University, Seoul, 08826, Republic of Korea; AIGENDRUG Co., Ltd., Seoul, 08758, Republic of Korea; Interdisciplinary Program in Artificial Intelligence, Seoul National University, Seoul, 08826, Republic of Korea; Department of Computer Science and Engineering, Seoul National University, Seoul, 08826, Republic of Korea

## Abstract

**Summary:**

Drug–target affinity (DTA) prediction is an important regression task for drug discovery, which can provide richer information than traditional drug–target interaction prediction as a binary prediction task. To achieve accurate DTA prediction, quite large amount of data are required for each drug, which is not available as of now. Thus, data scarcity and sparsity is a major challenge. Another important task is “cold-start” DTA prediction for unseen drug or protein. In this work, we introduce MixingDTA, a novel framework to tackle data scarcity by incorporating domain-specific pretrained language models for molecules and proteins with our MEETA (MolFormer and ESM-based Efficient aggregation Transformer for Affinity) model. We further address the label sparsity and cold-start challenges through a novel data augmentation strategy named GBA-Mixup, which interpolates embeddings of neighboring entities based on the guilt-by-association (GBA) principle, to improve prediction accuracy even in sparse regions of DTA space. Our experiments on benchmark datasets demonstrate that the MEETA backbone alone provides up to a 19% improvement of mean squared error over current state-of-the-art baseline, and the addition of GBA-Mixup contributes a further 8.4% improvement. Importantly, GBA-Mixup is model-agnostic, delivering performance gains across all tested backbone models of up to 16.9%. Case studies shows how MixingDTA interpolates between drugs and targets in the embedding space, demonstrating generalizability for unseen drug–target pairs while effectively focusing on functionally critical residues. These results highlight MixingDTA’s potential to accelerate drug discovery by offering accurate, scalable, and biologically informed DTA predictions.

**Availability and implementation:**

The code for MixingDTA is available at https://github.com/rokieplayer20/MixingDTA.

## 1 Introduction

Drug–target affinity (DTA) prediction is a fundamental task in drug discovery, enabling the quantification of interactions between small molecules and proteins. Recent advancements in deep learning, such as convolutional neural networks (CNNs), graph neural networks (GNNs) and transformer-based architectures, have demonstrated promising predictive power on benchmark datasets (Lim *et al.* 2021). Recently, methods incorporating multihead attention mechanisms (MHAs) (Vaswani *et al.* 2017) have gained attention for their ability to aggregate drug and target protein representations. MHA captures multiple dependencies between the components or “tokens” of drugs and targets simultaneously, allowing the model to focus on different aspects of their interaction. For instance, AttentionDTA (Zhao *et al.* 2023) uses cross-attention layers to align drug and protein embeddings, improving prediction accuracy in scenarios where affinity labels are abundant. While these regression-based methods provide insight beyond the binary relationship in ranking the therapeutic drugs with exact binding strengths, it requires more training data (Thafar *et al.* 2022), leading to the critical challenges of data scarcity and sparsity.

One of the primary challenges in DTA is data *scarcity*, which refers to the fact that only a small portion of drug–target pairs have been experimentally measured due to the high cost and complexity of wet-lab experiments. These results in missing affinity measurements for a large number of potential drug–target combinations, leaving significant gaps in the dataset (Supplementary Fig. S1). Data *sparsity* further describes the skewed nature of the observed affinity values within the regression spectrum. Many values cluster around a small region of specific range. Other remaining portions (e.g. extreme high or low affinities) remain underrepresented. For example, the affinity values in the DAVIS dataset are overpopulated in the range of [5.00, 7.19], accounting for 94.30% of the total values within the overall range of [5.00, 9.94] (Davis *et al.* 2011). Furthermore, 99.80% of the labels in the KIBA dataset are concentrated within the range [9.56, 17.20], which is a subset of the total [0.0, 17.20] (Tang *et al.* 2014). For this reason, these underrepresented portions form “sparse” regions of the label space. As highlighted by Pei *et al.* (2023), existing works often fail to address either the scarcity of measured data or the sparsity in the distribution of affinity values.

To mitigate the sparse drug–target relationships, there have been attempts to address the DTA problem using network-based approaches (Zeng *et al.* 2020, Thafar *et al.* 2022, Wang *et al.* 2024). Many studies, particularly those using drug–target bipartite graphs, capture local relationships via GNNs (Wang *et al.* 2024). Drugs and targets serve as nodes in these graphs, and the measured affinity corresponds to the edge weight. These approaches aim to learn from neighboring relations for improved predictions. However, relying on the drug–target bipartite graph introduces the crucial limitation that makes it incapable of predicting binding affinity of new drug or protein in cold start setting, since novel drugs or proteins lack connections in the training graph. Hence, there is a need to go beyond explicit neighborhood relations and enhance representational capacity through more flexible approaches.

To address these challenges, we propose MixingDTA, a novel deep learning-based framework designed to handle both data scarcity and sparsity using pretrained models and data augmentation approach inspired by network biology. MixingDTA framework consists of newly designed DTA architecture MEETA, augmented with our novel network-driven mixup technique GBA-Mixup. MEETA (MolFormer and ESM-based Efficient aggregation Transformer for Affinity) model efficiently incorporates pretrained language models for both molecules and proteins while replacing the computationally expensive MHA with attention-free aggregation (AFA) (Zhai *et al.* 2021) to improve efficiency.

A key component of MixingDTA is GBA-Mixup, a novel data augmentation method designed to address data sparsity. Rooted in the GBA principle of “associated biological entities share similar functions,” GBA-Mixup acts as a network biology-inspired data augmentation strategy. GBA-Mixup flexibly leverages the neighbor relations by constructing several DTA networks from different perspectives. From these DTA networks, where drug–target (D-T) pairs serve as nodes, GBA-Mixup generates “virtual” D–T pairs augmented by interpolating embeddings of related D–T pairs. For example, embeddings of pairs sharing common ligands or targets are mixed to create augmented training samples. This approach captures implicit relationships without relying solely on explicit graph edges, improving generalization to unseen D–T pairs and filling gaps in sparse regions of the DTA label space. Lastly, we integrate different perspectives of GBA-Mixup into a single meta-predictor to yield robust DTA prediction.

Together, GBA-Mixup and efficient pretrained model aggregation techniques enable MixingDTA to achieve state-of-the-art performance on benchmark datasets, particularly in challenging cold-start scenarios. Furthermore, GBA-Mixup’s model-agnosticity enhances performance across diverse existing models, demonstrating the consistent improvement of utilizing neighborhood information through mixup. Comprehensive case studies show how GBA-Mixup accurately interpolates embeddings of related D–T pairs, effectively focusing on critical residues and outperforming existing methods, even for unseen kinase–imatinib complexes.

## 2 Materials and methods

### 2.1 Overview of MixingDTA framework

**Figure 1.**
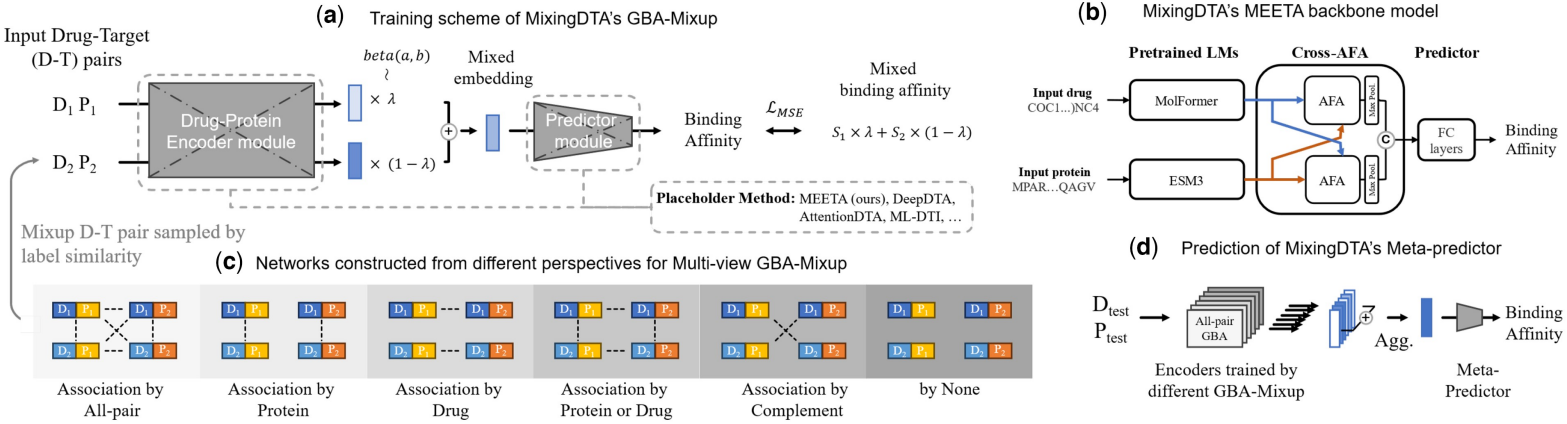
Overview of MixingDTA. (a) Two drug–target (D–T) pairs are provided as input, and a mixing ratio is sampled from a Beta distribution. This process generates new “virtual” D–T pairs with mixed embeddings. (b) The MEETA architecture integrates embeddings from pretrained language models for both drugs and proteins. It employs cross-AFA (attention-free aggregation) to efficiently aggregate representations. All components of the MEETA model after ESM3 and MolFormer are trained. (c) Neighboring D–T pairs are identified from multiple perspectives (e.g. by common drug or protein). Based on these perspectives, Mixup pair is sampled from the neighbors based on affinity label similarity. (d) Multiview integration combines embeddings from six GBA-Mixup–trained encoders, each capturing a different neighborhood perspective. These integrated embeddings are fed into the meta-predictor’s fully connected layers for final binding-affinity prediction.

MixingDTA is a novel DTA prediction framework that consists of three key components: a backbone model (e.g. MEETA), the GBA-Mixup augmentation strategy based on the GBA principle, and a multiview integration step (Fig. 1). This design enables MixingDTA to leverage biologically informed relationships while maintaining high computational efficiency and predictive accuracy. The details for each section can be found in following sections:

1. **MEETA** model (Fig. 1b): Section 2.2.
2. **GBA-Mixup** (Fig. 1c): Section 2.3.
3. **Multiview integration** of the trained encoders with meta-predictor (Fig. 1d): Section 2.3.2.

### 2.2 The MEETA backbone model

#### 2.2.1 Pre-trained language models

Our MEETA backbone model leverages two pretrained biomolecular language models, MolFormer (Ross *et al.* 2022) and ESM3 (Hayes *et al.* 2025), to extract meaningful representations of drugs and proteins:

MolFormer (Ross *et al.* 2022) is a transformer-based molecular language model designed for SMILES representations and pretrained in a self-supervised manner. It captures chemical properties and provides well-refined representations for diverse drug molecules. For MEETA, we utilized the MolFormer-XL version, ensuring comprehensive molecular encoding.

ESM3 (Hayes *et al.* 2025) is a transformer-based multimodal generative model trained across protein-related modalities, including sequence, structure, and function. Its masked language modeling pretraining spans a large corpus of protein data, enabling generalization across diverse protein families. For implementation, we used ESM3-open (1.4B), the smallest and fastest version publicly available. The version balances computational efficiency with competitive performance in protein representation learning.

These pretrained models form the foundation of MEETA, equipping MixingDTA with biologically informed embeddings for both drugs and proteins.

#### 2.2.2 Attention-free aggregation mechanism

Multihead cross-attention (MHA) (Vaswani *et al.* 2017) has been widely adopted for joint modeling of token representations from two distinct domains, including tasks such as DTA prediction. The output of multihead attention is defined as follows:


$$\text{MHA}(Q,K,V)=\text{Concat}({\text{head}}_{1},\ldots ,{\text{head}}_{H})W^{O},$$

where *H* is the number of attention heads, $W^{O}$ is a learned projection matrix, and each head is computed independently as follows:


$${\text{head}}_{i}=\text{softmax}\left(\frac{Q^{\left(i\right)}K^{{\left(i\right)}^{⊤}}}{\sqrt{d_{k}}}\right)V^{\left(i\right)},$$

where $Q^{\left(i\right)}=QW_{i}^{Q}$, $K^{\left(i\right)}=KW_{i}^{K}$, and $V^{\left(i\right)}=VW_{i}^{V}$ are the query, key, and value matrices for the *i*th head. Here, $W_{i}^{Q}$, $W_{i}^{K}$, and $W_{i}^{V}$ are learned projection matrices for the query, key, and value in the *i*th head, with $\sqrt{d_{k}}$ scaling the dot-product by the square root of the key’s dimensionality ($d_{k}$) to stabilize gradients.

While powerful, the computational burden associated with its all-pairwise dot products in traditional MHA mechanisms limits scalability. Specifically, the quadratic complexity of these operations $O(T^{2})$, with respect to sequence length *T*, presents challenges for handling long sequences. This often forces MHA-based models to truncate protein and molecular sequences, an approach that is biologically irrational for DTA prediction, where retaining all substructural and subsequential information is critical for accurately capturing drug–target binding interactions.

The computational overhead of MHA’s dot-product has led to the development of alternative cross-attention modules with linear complexity (Katharopoulos *et al.* 2020, Zhai *et al.* 2021). Inspired from the approaches, we adopted an AFA mechanism for cross-attention that eliminates the need for dot products. The term “attention-free” distinguishes this absence of the dot product from original attention mechanism, first adopted by Zhai *et al.* (2021). This method relies on efficient element-wise operations, reducing computational complexity to linear O(T) scale. This improvement enables the model to process longer sequences without sacrificing computational efficiency or accuracy, ensuring the inclusion of all relevant drug and protein features. The AFA mechanism is defined as follows:


$$\text{AFA}(Q,K,V)=\sigma_{q}(Q^{\left(0\right)})⊙\frac{\sum_{t^{\prime }=1}^{T}\;\text{exp}\;(K_{t^{\prime }}^{\left(0\right)})⊙V_{t^{\prime }}^{\left(0\right)}}{\sum_{t^{\prime }=1}^{T}\;\text{exp}\;(K_{t^{\prime }}^{\left(0\right)})},$$

where ⊙ denotes the Hadamard (element-wise) product, $t^{\prime }$ represents the token position in the sequence, and $\sigma_{q}$ is the sigmoid activation function applied to the query *Q*. $Q^{\left(0\right)}=QW^{Q}$, $K^{\left(0\right)}=KW^{K}$, $V^{\left(0\right)}=VW^{V}$ are the query, key, and value matrices for the AFA module, where $W^{Q}$, $W^{K}$, and $W^{V}$ are learned projection matrices. Only a single head is applied for the AFA module to achieve the computational efficiency.

For example, when modeling the interaction between a drug and a protein sequence for the DTA task, the tokenized representation of drug and protein can be represented as $\left\{d_{1},d_{2},\ldots ,d_{T_{d}}\right\}$, where $T_{d}$ is the number of drug tokens and$\left\{p_{1},p_{2},\ldots ,p_{T_{p}}\right\}$, where $T_{p}$ is the number of protein tokens.

For a drug token $d_{t}$, the aggregated representation $Y_{d_{t}}$ is computed with respect to the protein tokens $\left\{p_{t^{\prime }}\right\}$ using the AFA mechanism:


$$Y_{d_{t}}=\sigma_{q}(Q_{d_{t}})⊙\frac{\sum_{t^{\prime }=1}^{T_{p}}\;\text{exp}\;(K_{p_{t^{\prime }}})⊙V_{p_{t^{\prime }}}}{\sum_{t^{\prime }=1}^{T_{p}}\;\text{exp}\;(K_{p_{t^{\prime }}})},$$

with the resulting $Y_{d_{t}}$ is the aggregated representation of the drug token $d_{t}$ relative to all protein tokens $\left\{p_{t^{\prime }}\right\}$. This computation is repeated for each drug token $d_{t}$, resulting in a sequence of cross-attended drug representations $\left\{Y_{d_{1}},Y_{d_{2}},\ldots ,Y_{d_{T_{d}}}\right\}$. After retrieving all $T_{d}$ representations for the tokens of the input drug, maximum value within each region is selected as the output to retrieve the final representation of the drug (Ranzato *et al.* 2007).

This simplified approach maintains the essential properties of cross-attention while drastically improving efficiency. The method ensures computational and memory efficiency by using element-wise operations. It enables the model to effectively integrate token representations from drug and protein without compromising performance or scalability.

Especially due to its linear complexity O(T), it allows for the processing of longer sequences. This is critical for tasks requiring high-resolution representations of molecular and protein structures. Unlike models that truncate sequences, our approach’s efficiency allows preservation of full drug and protein sequences along with their biological contexts, enabling more accurate modeling of their binding.

### 2.3 GBA-Mixup

Along with the MEETA model, we propose GBA-Mixup, a novel adaptation of Mixup rooted in the GBA principle of network biology. Originally, Mixup is a data augmentation strategy where synthetic data points are generated by interpolating between pairs of original samples using a weighted average (Zhang *et al.* 2018, Yao *et al.* 2022). The Mixup ratio is typically sampled from a Beta distribution, ensuring a flexible and randomized interpolation process. For regression tasks, including DTA prediction, both the input features and the affinity labels are interpolated to create new synthetic data points. It effectively enhances generalization performance and reducing overfitting. Naive Mixup technologies attempt to interpolate between all-pairs of training data points. However, our core idea of the GBA-Mixup approach is to leverage the guilt-by-association (GBA) principle, which states that “associated proteins share similar functions.”

In order to incorporate network biology context into Mixup, we define each drug–target pair as a node in the DTA problem, and the affinity value becomes the node’s label. Then, we define the neighborhood relationships based on shared components (e.g. drug or target) to incorporate network structures into the data augmentation process. Various networks can be constructed depending on how edges are assigned, leading to diverse pairing distributions. Lastly, the edge weights between the neighbors are calculated using the node label similarity, later utilized as probabilities for Mixup pair sampling.

#### 2.3.1 Sampling D–T Mixup pairs

##### 2.3.1.1 Different perspectives of neighborhood

Each D–T pair can be represented as a node in the DTA data space. Six distinct network scenarios can be defined based on the type of shared components when establishing the connections between the D–T nodes (Fig. 1c):

1. **None:** No connections; nodes remain isolated without any GBA-Mixup.
2. **All-pair:** A naive Mixup scenario where all nodes are connected inside the training set, with edge weights reflecting the similarity of their binding affinities.
3. **Drug:** Only the nodes sharing the same drug are connected.
4. **Protein:** Only the nodes sharing the same target are connected.
5. **Protein or drug:** Nodes are connected if they share either a drug or a target.
6. **Complement:** The complement of the “protein or drug” scenario, where nodes are connected only if they share neither a drug nor a target.

After the networks are constructed, the Mixup D–T pairs are sampled from neighborhoods based on edge weights, following the procedure described below.

##### 2.3.1.2 Sampling Mixup pairs

To further refine the D–T pair sampling of the Mixup process, GBA-Mixup employs a weighted sampling strategy based on the similarity of binding affinities. Nodes with similar affinity values are assigned higher edge weights, increasing their probability of selection as Mixup pairs. This weighted approach ensures that the synthetic data points generated through Mixup better reflect the underlying biological relationships between drugs and targets, compared to random pair sampling (Zhang *et al.* 2018, Yao *et al.* 2022).

Once node pairs are selected based on their neighborhood relationships and affinity-weighted probabilities, synthetic samples are generated by interpolating their input features and labels. Let $\left(x_{i},y_{i}\right)$ and $\left(x_{j},y_{j}\right)$ represent two selected nodes with features *x* and labels *y*. The interpolated sample $\left(x_{\text{mix}},y_{\text{mix}}\right)$ is computed as follows:


$$x_{\text{mix}}=\lambda x_{i}+(1-\lambda )x_{j},\;y_{\text{mix}}=\lambda y_{i}+(1-\lambda )y_{j},$$

where λ∼Beta(α,α), and α is a hyperparameter controlling the degree of interpolation. Using the six GBA-Mixup scenarios, we construct six networks and train the MEETA model independently on each network.

#### 2.3.2 GBA-Mixup training

The GBA-Mixup training process consists of two sequential steps: (i) Training multiple MEETA backbone models using distinct GBA perspectives, then (ii) aggregating the latent embeddings and predictions from these models into a meta-predictor to learn multiview representations.

##### 2.3.2.1 Step 1: Training with GBA perspectives

Using the six predefined GBA-Mixup scenarios—none, all-pair, drug, protein, protein or drug, and complement—we constructed augmented networks to capture diverse neighborhood relationships in the DTA embedding space. These diversity of the neighborhood definitions allows the MEETA backbone model to learn embeddings tailored to specific network structures. Independent training was performed for each scenario, resulting in six separate MEETA models.

##### 2.3.2.2 Step 2: Meta-predictor training

Following the training of the six base models, embeddings and predictions were extracted for all D–T pairs. These outputs were summed to form a unified representation for each pair, capturing complementary information from the six network perspectives.

The aggregated representations were then input into a meta-predictor, which shares the same fully connected layer architecture as the MEETA prediction layers. The meta-predictor is trained to utilize the multiview representations. It effectively integrates insights from the six GBA scenarios to enhance its predictive accuracy.

This comprehensive framework positions GBA-Mixup as a biologically informed augmentation tool for DTA prediction models, achieving a balance between biological relevance and model’s generalizability.

### 2.4 Dataset and metrics

#### 2.4.1 Datasets

Two benchmark datasets, DAVIS (Davis *et al.* 2011) and KIBA (Tang *et al.* 2014), were used to conduct experiments on MixingDTA and the baseline models. Both datasets from TDC (tdcommons.ai/multi_pred_tasks/dti, accessed 30 October 2024) (Huang *et al.* 2021) comprise protein–ligand pairs and continuous binding affinities. Ligands are represented using SMILES, while proteins are given as amino acid sequences. The binding affinity can include values such as the dissociation constant ($K_{d}$), half-maximal inhibitory concentration (IC50), or inhibition constant ($K_{i}$). DAVIS dataset contains 25 772 pairs, with 68 drugs and 379 proteins. The labels range from [5.00, 9.94]. Meanwhile, the KIBA dataset contains 117 657 pairs, with 2068 drugs and 229 proteins. We followed well-established data processing procedure for stable training. For DAVIS dataset, binding-affinity values are first harmonized using the max_affinity function and then converted to log-scale ($pK_{d}$). KIBA labels—known as KIBA scores—are derived from underlying bioactivity values related to IC50, $K_{i}$, and $K_{d}$, and range from [0.0, 17.20]. The statistics of each dataset is shown in Supplementary Table S1.

#### 2.4.2 Experimental setup

We conducted experiments using a five-fold cross-validation to compare the performance of baseline models and MixingDTA on the DAVIS and KIBA datasets. The train:valid:test ratio was set to 6:2:2. Additionally, while some previous DTA studies employed a truncation setting that uses only partial strings of drugs or proteins, this comparative experiment utilizes full strings without truncation. Notably, CSCo-DTA cannot perform cold start experiments due to the need for artificially constructing a bipartite graph. This is explained in Supplementary Methods S3.1. The models are trained based on mean squared error (MSE) loss.

The hyperparameters of MixingDTA, settings for the model-agnostic experiments and details of experiments under random split are provided in Supplementary Table S2. The multiview integration training in Stage 2 follows the same setup as also described in Supplementary Table S2.

#### 2.4.3 Evaluation metrics

We assessed the performance of MixingDTA and baseline models using a comprehensive suite of evaluation metrics: MSE, concordance index (CI), $r_{m}^{2}$, and the area under the precision–recall curve (AUPR). Detailed explanations, including the formulas, are provided in Supplementary Methods S3.2.

MSE quantifies the average squared difference between true and predicted values, providing insight into the model’s prediction accuracy. The CI evaluates the ranking accuracy of predictions, measuring whether higher predicted scores align with higher true values. $r_{m}^{2}$ is designed for external validation such as test datasets. It assesses how well the model explains the relationship between the actual and predicted values. It balances squared correlation coefficients with and without intercepts to provide a robust measure of predictive power. AUPR highlights the model’s ability to distinguish relevant interactions, especially in imbalanced datasets. Thresholds of 7 for the DAVIS dataset and 12.1 for the KIBA dataset were used, following well-established benchmarks (Öztürk *et al.* 2018).

The other evaluation indicators are described for further analysis. These include additional widely used metrics such as root mean squared error (RMSE), mean absolute error, $R^{2}$, Pearson’s correlation coefficient and Spearman’s correlation coefficient. Detailed comparisons can be found in Supplementary Tables S3 and S4. Additionally, the details of the similarity measurement used in the interpolation case study can be found in Supplementary Methods S3.3.

## 3 Results and discussion

### 3.1 Performance comparison of DTA models

#### 3.1.1 Random split (warm start)

In this study, we evaluated the predictive performance of multiple DTA models using warm start settings on two widely used benchmark datasets: DAVIS and KIBA. Both the training and test sets include overlapping drugs and proteins under this configuration. Every molecule or sequence in the test set has been encountered during training.

Our proposed framework named MixingDTA consistently demonstrated better performance compared to several state-of-the-art models, including DeepDTA (Öztürk *et al.* 2018), GraphDTA (Nguyen *et al.* 2021), AttentionDTA (Zhao *et al.* 2023), ML-DTI (Yang *et al.* 2021), MGraphDTA (Yang *et al.* 2022), CSCo-DTA (Wang *et al.* 2024), and MD-CT-DTA (Zhu *et al.* 2024). Table 1 presents the detailed comparison of evaluation metrics across both datasets. Detailed information on related works can be found in Supplementary Methods S3.4.

**Table 1.** Performance on DAVIS and KIBA datasets with warm start setting.^a^

	DAVIS				KIBA			
	MSE (↓)	CI (↑)	$r_{m}^{2}$ (↑)	AUPR (↑)	MSE (↓)	CI (↑)	$r_{m}^{2}$ (↑)	AUPR (↑)
CSCo-DTA	0.283 (0.0177)	0.862 (0.0050)	0.559 (0.0171)	0.605 (0.0235)	0.203 (0.0131)	0.852 (0.0052)	0.698 (0.0159)	0.745 (0.0107)
MD-CT-DTA	0.275 (0.0072)	0.857 (0.0061)	0.555 (0.0244)	0.623 (0.0147)	0.237 (0.0162)	0.825 (0.0089)	0.626 (0.0294)	0.721 (0.0161)
DeepDTA	0.243 (0.0140)	0.865 (0.0088)	0.614 (0.0219)	0.653 (0.0152)	0.190 (0.0053)	0.865 (0.0031)	0.691 (0.0083)	0.772 (0.0069)
GraphDTA	0.241 (0.0106)	0.869 (0.0076)	0.632 (0.0237)	0.667 (0.0124)	0.177 (0.0033)	0.868 (0.0025)	0.733 (0.0072)	0.779 (0.0050)
ML-DTI	0.231 (0.0092)	0.874 (0.0056)	0.630 (0.0302)	0.655 (0.0140)	0.186 (0.0040)	0.867 (0.0032)	0.706 (0.0100)	0.774 (0.0072)
MGraphDTA	0.217 (0.0150)	0.879 (0.0075)	0.673 (0.0235)	0.676 (0.0148)	0.148 (0.0029)	**0.894 (0.0015)**	0.775 (0.0103)	**0.807 (0.0026)**
AttentionDTA	0.215 (0.0090)	0.879 (0.0066)	0.663 (0.0284)	0.672 (0.0084)	0.167 (0.0027)	0.880 (0.0017)	0.732 (0.0116)	0.788 (0.0032)
MEETA	0.181 (0.0096)	0.889 (0.0099)	0.711 (0.0217)	0.716 (0.0138)	0.153 (0.0019)	0.881 (0.0028)	0.768 (0.0150)	0.792 (0.0056)
MixingDTA	**0.167 (0.0095)**	**0.906 (0.0046)**	**0.745 (0.0165)**	**0.730 (0.0152)**	**0.142 (0.0013)**	0.880 (0.0017)	**0.780 (0.0129)**	0.798 (0.0023)

a The results of five-fold cross-validation are provided. Best performances in bold and second-best underlined.

Among the baseline models, our backbone architecture MEETA achieved competitive performance even before applying the GBA-Mixup augmentation. These results showcase the effectiveness of our architectural design when combined with state-of-the-art pretrained models.

MixingDTA, equivalent to MEETA enhanced with GBA-Mixup, showed improved performance, with reduction in the MSE on both datasets. This improvement underscores the effectiveness of GBA-Mixup in leveraging the relationships between protein-sharing and drug-sharing data points to diversify training. To summarize the improvements, the scatter plots for the datasets (Supplementary Fig. S2) illustrate the distribution of the predicted values against true affinity values for each dataset. Furthermore, ablation studies of each GBA perspective for GBA-Mixup demonstrates that aggregation of all six scenarios leads to the optimal performance. This includes the Mixup by “Complement” and “None” cases, which we interpret as bringing diversity to the representation space which leads to performance improvement (Supplementary Figs S3 and S4, Supplementary Tables S5–S8).

To conduct more comprehensive performance comparison, we performed additional evaluations. Specifically, we evaluated the models under the random split with two datasets: BindingDB Kd (Liu *et al.* 2006) and PDBbind Refined (Liu *et al.* 2014). MixingDTA achieves the best performance in all cases (Supplementary Tables S9 and S10).

#### 3.1.2 Blind split (cold start)

To further assess the generalization capability of the DTA models, we evaluated their performance under the cold-start (blind split) setting using the DAVIS dataset. In this more challenging scenario, the training and test sets do not share any overlapping drugs or proteins, simulating real-world conditions where models are required to predict the affinity of novel compounds or previously unseen targets. To note, CSCo-DTA (Wang *et al.* 2024), a D–T bipartite network-based model, is incapable of predicting drugs or targets unseen during training phase. Hence, these baselines were not included in the results for this section. The results are summarized in Table 2, with MixingDTA achieving superior performance compared to all other models. This was observed under both cold drug and cold protein settings.

**Table 2.** Performance on DAVIS datasets with drug- and target-cold start (blind split) setting.^a^

	DAVIS (unseen/cold target)				**DAVIS (unseen/cold drug**)			
	MSE (↓)	CI (↑)	$r_{m}^{2}$ (↑)	AUPR (↑)	MSE (↓)	CI (↑)	$r_{m}^{2}$ (↑)	AUPR (↑)
DeepDTA	0.358 (0.0233)	0.807 (0.0129)	0.407 (0.0182)	0.508 (0.0331)	0.714 (0.0533)	0.644 (0.0422)	0.065 (0.0403)	0.202 (0.0627)
AttentionDTA	0.330 (0.0089)	0.823 (0.0062)	0.413 (0.0169)	0.531 (0.0225)	0.702 (0.0640)	0.666 (0.0342)	0.108 (0.0302)	0.257 (0.0263)
ML-DTI	0.350 (0.0167)	0.813 (0.0070)	0.408 (0.0229)	0.518 (0.0286)	0.688 (0.0907)	0.672 (0.0321)	0.111 (0.0582)	0.269 (0.0790)
MGraphDTA	0.335 (0.0161)	0.820 (0.0077)	0.412 (0.0249)	0.539 (0.0301)	0.656 (0.0944)	0.724 (0.0185)	0.190 (0.0459)	0.428 (0.0759)
MD-CT-DTA	0.466 (0.0216)	0.767 (0.0132)	0.246 (0.0197)	0.405 (0.0191)	0.547 (0.0581)	0.706 (0.0298)	0.239 (0.0600)	0.501 (0.1280)
GraphDTA	0.484 (0.0172)	0.737 (0.0084)	0.228 (0.0100)	0.353 (0.0108)	**0.538 (0.0575)**	0.728 (0.0182)	0.228 (0.0641)	0.329 (0.1259)
MEETA	0.244 (0.0099)	0.857 (0.0032)	0.531 (0.0239)	0.631 (0.0131)	0.573 (0.0288)	0.734 (0.0066)	0.238 (0.0130)	0.519 (0.0181)
MixingDTA	**0.231 (0.0042)**	**0.874 (0.0017)**	**0.567 (0.0144)**	**0.634 (0.0105)**	**0.538 (0.0123)**	**0.754 (0.0048)**	**0.258 (0.0072)**	**0.523 (0.0110)**

a Mean and standard deviation of five-fold cross-validation are provided. Best performances in bold and second-best underlined.

Under the cold protein setting, MixingDTA achieved outstanding predictive metrics with a CI of 0.87 and an MSE of 0.23, representing a significant improvement over the second-best model (CI = 0.82, MSE = 0.33). The improvement in this setting underscores MixingDTA’s potential to predict interactions with novel biological targets, which is critical for drug discovery applications involving emerging diseases or poorly characterized proteins.

In the more challenging cold drug scenario, where the model predicts affinities for unseen drug structures, MixingDTA achieved CI of 0.75 and MSE of 0.54, surpassing all competing models by a significant margin (paired *t*-test *P*<.05) in CI, $r_{m}^{2}$, and AUPR. These results underline MixingDTA’s ability to generalize across unseen chemical spaces, an essential attribute for novel drug discovery tasks.

In summary, the cold-start evaluation reveals the remarkable performance of MixingDTA across both cold drug and cold target settings. By using GBA-Mixup, MixingDTA can harness essential information from six different types of potential D–T pair relationships. This establishes it as a highly effective solution for real-world DTA prediction tasks. It is well suited for making predictions involving novel compounds or unexplored protein targets.

### 3.2 Evaluation of the generalizability and effectiveness of GBA-Mixup across models

The proposed GBA-Mixup is a biologically informed method. It is designed to incorporate context-driven data augmentation. Its flexibility and adaptability make it inherently model-agnostic, enabling integration with a wide range of DTA prediction architectures. To demonstrate the versatility of GBA-Mixup, we evaluated its impact on several state-of-the-art DTA models. These models ranged from convolutional neural network-based architectures to attention-based models.

The results in Table 3 demonstrate the consistent and significant performance improvements achieved by incorporating GBA-Mixup into diverse model architectures. Notably, all models exhibited improvements in every evaluation metrics. The most notable improvement was observed in MSE, with reductions of up to −16.9%. It indicates the consistent improvement across all evaluated benchmarks. Especially GBA-Mixup improved both convolutional models (e.g. DeepDTA) and attention-based models (e.g. AttentionDTA), which underscores its architecture-independent nature. For instance, the integration of GBA-Mixup reduced MSE for DeepDTA by −12.8% and for AttentionDTA by −13.6%. These results emphasize its broad applicability to diverse DTA frameworks.

**Table 3.** Performance of baseline models and their performance gain with GBA-Mixup applied on DAVIS dataset (warm start).^a^

	**MSE (** ↓ **)**	**CI (** ↑ **)**	$r_{m}^{2}$ **(**↑**)**	**AUPR (** ↑ **)**
DeepDTA	0.24 (0.014)	0.87 (0.009)	0.61 (0.022)	0.65 (0.015)
w/GBA-Mixup	**0.21 (0.011)**	**0.89 (0.005)**	**0.68 (0.026)**	**0.68 (0.018)**
	(−12.8%)	(+2.5%)	(+10.8%)	(+4.8%)
ML-DTI	0.23 (0.008)	0.87 (0.006)	0.63 (0.030)	0.65 (0.014)
w/GBA-Mixup	**0.19 (0.008)**	**0.90 (0.007)**	**0.70 (0.020)**	**0.69 (0.013)**
	(−16.9%)	(+2.3%)	(+11.5%)	(+5.2%)
AttentionDTA	0.22 (0.009)	0.88 (0.007)	0.66 (0.028)	0.67 (0.008)
w/GBA-Mixup	**0.19 (0.012)**	**0.90 (0.005)**	**0.71 (0.026)**	**0.71 (0.024)**
	(−13.6%)	(+2.0%)	(+7.1%)	(+5.6%)
MEETA (ours)	0.18 (0.010)	0.89 (0.010)	0.71 (0.022)	0.72 (0.014)
w/GBA-Mixup	**0.17 (0.010)**	**0.91 (0.005)**	**0.75 (0.017)**	**0.73 (0.015)**
	(−7.7%)	(+1.9%)	(+4.8%)	(+2.0%)

a Mean and standard deviation of five-fold cross-validation are provided. The bold texts indicate that performance improvement was achieved.

The model-agnostic experiments were also conducted in cold-start settings and consistently showed performance improvements regardless of the backbone model. The results of this experiment can be found in Supplementary Table S11 and Supplementary Methods S3.5.

We interpret these universal performance gains as a result of leveraging protein-sharing or drug-sharing data points during training. GBA-Mixup generates augmented samples that better capture the underlying potential interactions. This biological context-driven augmentation shows effectiveness in mitigating overfitting and improving generalization, particularly in DTA’s sparse-data scenarios. These findings establish GBA-Mixup as a valuable enhancement to existing DTA prediction frameworks. This can offer universal benefits without requiring architecture-specific modifications.

### 3.3 Efficiency and accuracy of MEETA’s AFA

We further conducted an ablation study to evaluate the effectiveness of different cross-attention mechanisms on the MEETA model in aggregating token representations from molecular and protein language models. The compared aggregation method is widely adopted MHA along with MEETA’s AFA. Summarized in Table 4, the prediction performance reveals the performance and resource efficiency of AFA in DAVIS dataset. Specifically, MEETA-AFA demonstrated the higher predictive performance across the evaluation metrics against MEETA-MHA. For instance, MEETA-AFA achieved a CI of 0.89 and an MSE of 0.18. In contrast, MHA shows lower performance with a CI of 0.87 and an MSE of 0.21. These findings highlight the capability of AFA to effectively aggregate the contextual representations of molecular and protein embeddings.

**Table 4.** Performance of MEETA’s AFA, MHA variants applied on DAVIS dataset (warm start).^a^

	**MSE (** ↓ **)**	**CI (** ↑ **)**	$r_{m}^{2}$ **(**↑**)**	**AUPR (** ↑ **)**
MEETA-MHA	0.21 (0.013)	0.87 (0.008)	0.66 (0.023)	0.69 (0.023)
MEETA-AFA	**0.18 (0.010)**	**0.89 (0.010)**	**0.71 (0.022)**	**0.72 (0.014)**

a Mean and standard deviation of five-fold cross-validation are provided. The bold texts indicate that performance improvement was achieved.

As discussed prior in Section 2, AFA achieves computational complexity of O(T), compared to the $O(T^{2})$ complexity of MHA due to its avoidance of the computationally expensive dot product operation. Numerical details regarding memory usage are provided in Supplementary Fig. S5.

Overall, AFA achieves the balance between prediction performance and efficiency. It demonstrates the highest performance in DTA prediction while achieving computational simplicity. The computational requirements of AFA make it suitable for large-scale DTA studies and integration into resource-constrained environments. The incorporation of AFA as the cross-attention mechanism in DTA models demonstrates its significant value. It provides an efficient and effective approach to aggregating complex representations.

### 3.4 Case study on GBA-Mixup interpolation between drugs and targets

**Figure 2.**
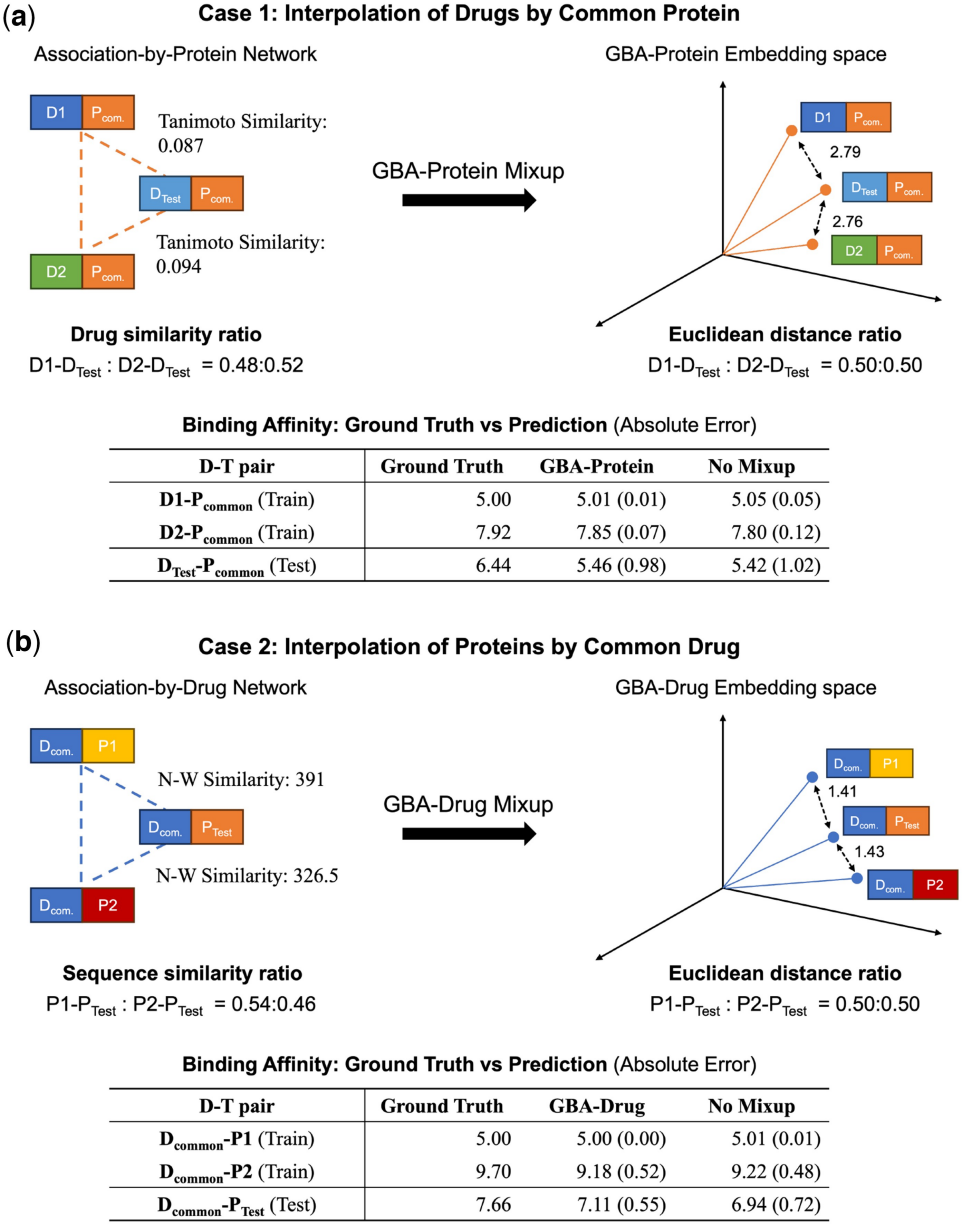
Case study on GBA-Mixup’s interpolation between drugs and targets on DAVIS dataset. Despite performing Mixup based on label similarity with the neighboring connections, the model successfully constructed an embedding space that reflects the sequence similarity. The IDs for each drug and protein are detailed in Supplementary Table S10. (a) In the drug interpolation scenario, three drugs ($D_{1}$, $D_{2}$, and $D_{\text{Test}}$) share a common protein ($P_{\text{com}.}$). The Tanimoto similarity scores between $D_{1}$–$D_{\text{Test}}$ and $D_{2}$–$D_{\text{Test}}$ are 0.087 and 0.094, respectively. Embeddings generated by the MEETA model trained with GBA-Protein Mixup demonstrate that embedding-space distances closely align with Tanimoto similarity ratios, effectively capturing chemical similarities. The GBA-Protein Mixup model also achieves lower predictive error compared to the baseline MEETA model without Mixup. (b) In the target interpolation scenario, three proteins ($P_{1}$, $P_{2}$, and $P_{\text{Test}}$) share a common drug ($D_{\text{com}.}$). The MEETA model trained with GBA-Drug Mixup aligns embedding-space distances with N–W sequence similarity scores. These two cases reveal the ability of GBA-Mixup to capture domain-specific relationships for both drugs and proteins. N-W: Needleman–Wunsch.

To illustrate the effectiveness of GBA-Mixup, we conducted two detailed case studies involving interpolation between drugs and targets. In both cases, we tried to illustrate how GBA-Mixup facilitates meaningful interpolation in the D–T embedding space and improves DTA predictions. We present two scenarios: drug interpolation with the common protein in Fig. 2a and target interpolation with the common drug in Fig. 2b. Importantly, the interpolated D–T pairs involving $D_{\text{Test}}$ and $P_{\text{Test}}$ belong to the test set and were not seen during training to confirm the zero-shot generalizability of our approach.

In the drug interpolation scenario, three drugs—$D_{1}$, $D_{2}$, and $D_{\text{Test}}$—bind to the same protein, $P_{\text{com}.}$. Tanimoto similarities between $D_{1}$–$D_{\text{Test}}$ and $D_{2}$–$D_{\text{Test}}$ were 0.087 and 0.094, respectively. The MEETA model trained with GBA-Protein Mixup closely mirrored these similarity ratios in the embedding-space distances. As shown in Fig. 2a, the test point $\left(D_{\text{Test}},P_{\text{com}.}\right)$ is embedded in a position that reflects its structural similarity to both $D_{1}$ and $D_{2}$. Notably, the GBA-Mixup model achieved lower prediction error than the non-Mixup baseline, indicating that interpolating between chemically similar drugs contributes to more robust representation learning.

In the target interpolation scenario (Fig. 2b), three proteins—$P_{1}$, $P_{2}$, and $P_{\text{Test}}$—share a common drug, $D_{\text{com}.}$. Needleman–Wunsch (N–W) alignment similarity was used to quantify sequence similarity. Once again, the ratios of sequence similarity between pairs closely matched the Euclidean distances in the embedding space when using GBA-Drug Mixup. The test pair $\left(D_{\text{com}.},P_{\text{Test}}\right)$, unseen during training, was also accurately positioned relative to $P_{1}$ and $P_{2}$. Similar results were obtained with the KIBA dataset (Supplementary Fig. S6). These results highlight the interpretability of our Mixup process, with interpolated points reflecting realistic transitions between biologically or chemically related entities, rather than creating arbitrary or biologically unrealistic hybrids.

To further support interpretability, we performed a PCA-based visualization of the embedding space. As shown in Supplementary Fig. S7, the original space without Mixup exhibits noticeable sparsity, especially in underrepresented regions. In contrast, the GBA-Mixup method produces interpolated embeddings that densely populated these sparse zones, forming a more continuous and structured latent space. This support that GBA-Mixup meaningfully augments the space with biologically informed synthetic data, improving both coverage and generalization.

Overall, these results support that GBA-Mixup captures domain-specific similarities (chemical and sequence-based), constructs a well-populated embedding space, and significantly enhances generalizability by generating virtual training samples in data-scarce regions. Further experimental details and similarity-to-distance ratios are provided in Supplementary Methods S3.3 and Supplementary Tables S12 and S13.

### 3.5 Case study on binding site awareness of MixingDTA’s AFA mechanism

Despite not providing any pocket information or 3D structural inputs, we investigated how effectively MixingDTA can localize key binding (pocket) residues in a purely data-driven manner. Specifically, we examined whether the cross-AFA mechanism in MixingDTA focuses on the relevant residues for D–T interactions. This is then compared with the performance of AttentionDTA, MEETA-MHA, and MEETA-AFA.

Including our MEETA framework, the cross-AFA step produces integrated D–T representations like most attention-based DTA prediction models. Following this step, a token-wise max-pooling operation is performed on the protein residues. Because each of the 256 embedding dimensions “votes” for the residue that produces the maximum activation, we can backtrack to see which residue contributed most strongly. For instance, if a given residue contributes to 68 out of 256 dimensions, we count 68 max-pool occurrences for that residue. By analyzing these max-pooled residue counts, we obtain a rough mapping of which residues the model focuses on to the final representation—effectively inferring a data-driven “binding site” without explicit structural guidance.

To compare the quality of the predicted binding sites, we conducted a zero-shot case study on imatinib, a well-known kinase inhibitor. Specifically, we retrieved 20 cocrystal structures from the Protein Data Bank (PDB) where imatinib is bound to various kinases, along with the binding residues (within 4 Å from imatinib) organized by Lee *et al.* (2025). The full list is organized in Supplementary Table S14. Notably, neither imatinib nor these specific kinases were included in our training set, ensuring an unbiased evaluation.

We calculated the overlap between true binding residues and the residues prioritized by each method (AttentionDTA, MEETA-MHA, MEETA-AFA, and MixingDTA). As summarized in Supplementary Table S15, MixingDTA consistently achieved superior balanced metrics, including Matthews correlation coefficient, F1-score, and average precision. These results suggest that integrating GBA with AFA not only improves regression performance but also helps the model focus more precisely on the binding site.

**Figure 3.**
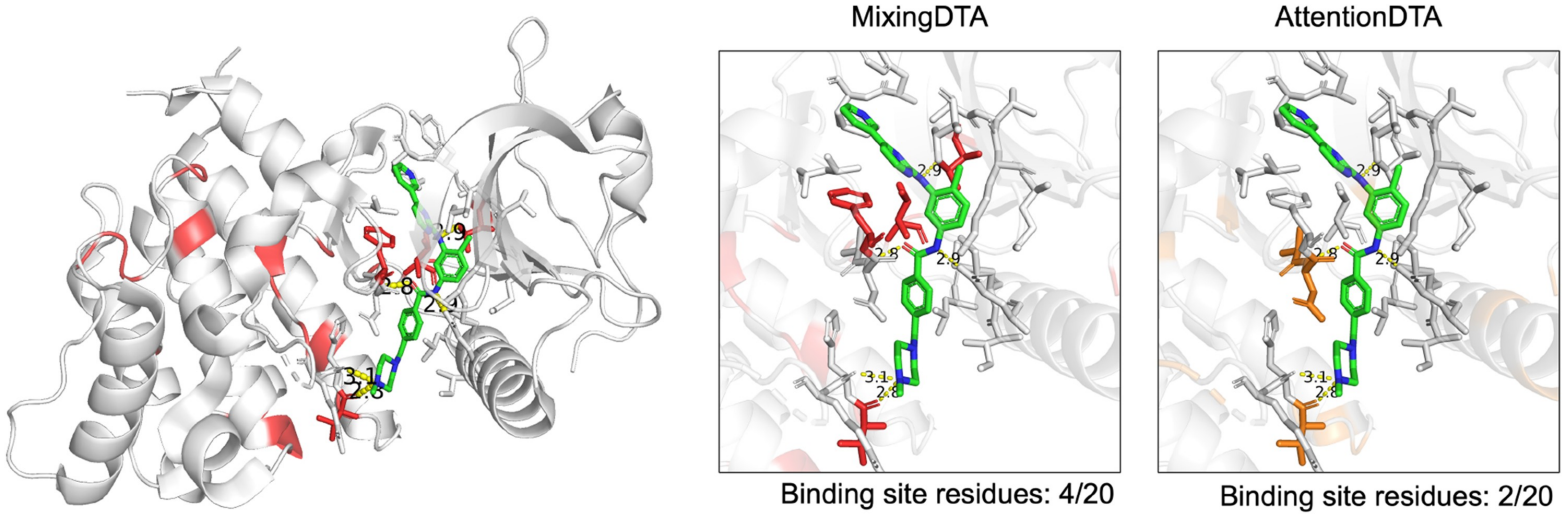
Visualization of top-20 focused residues (max-pooled residues) and the actual binding site of imatinib with human DDR1 Kinase domain (PDB: 4BKJ). MixingDTA focuses more on the binding site, with four residues among 20 including two hydrogen bondings, compared to two of AttentionDTA model. Binding sites are marked in sticks and hydrogen bondings are marked in yellow dashes, annotated with interaction distances. Max-pooled residues of MixingDTA and AttentionDTA are marked in red and orange, respectively. The 3D visualizations in this figure were generated using PyMOL.

As a representative example, we visualized the predicted binding site for imatinib bound to the Human DDR1 kinase domain (PDB ID: 4BKJ) (Fig. 3). The overlay of our top-20 predicted residues with the actual ligand-binding region of 20 residues (within 4 Å) indicates that MixingDTA’s AFA mechanism successfully highlights four critical side chains. The chains include hydrogen bonding sites, which closely match the experimentally resolved binding site compared to two of AttentionDTA’ multihead attention module.

Overall, these findings demonstrate that MixingDTA—despite the absence of explicit structural or pocket input—accurately identifies functionally important residues. The combination of pretrained embeddings, attention-free aggregation, and GBA-based data augmentation collectively enhances the model’s capacity to locate key ligand-binding sites in a challenging zero-shot setting.

## 4 Conclusion

In this work, we proposed a novel framework MixingDTA along with its components MEETA and GBA-Mixup, designed to address the DTA task’s challenges of data scarcity and sparsity. Domain-specific pretrained embeddings, a computationally efficient AFA mechanism, and the innovative GBA-Mixup strategy are employed to construct our framework. By the design, MixingDTA effectively captures implicit relationships between drugs and targets without relying on explicit graph topologies. As a result, our framework achieved state-of-the-art performance on benchmark datasets, particularly excelling in cold-start scenarios where unseen drugs and proteins were introduced. Furthermore, the model-agnostic design of GBA-Mixup ensures that performance improvements are consistent across CNN and transformer architectures. In comprehensive case studies, we demonstrated the framework’s ability to interpolate embeddings effectively. MixingDTA was able to highlight critical binding site residues as shown in experiments with DDR1 and imatinib. Additionally, it outperformed existing method in zero-shot binding site prediction tasks.

Despite these achievements, there are areas for further improvement. Notably, the model’s performance in cold-drug settings is yet to be satisfactory because performances of all models tested are below 0.5 in $r_{m}^{2}$. This underscores the difficulty of cold-drug split testing, which remains an open problem in the DTA field. Furthermore, categorization of GBA scenarios into more specific biochemical or pharmaceutical contexts could enhance the model’s predictive power.

In summary, MixingDTA demonstrates the effectiveness of combining pretrained embeddings, efficient aggregation mechanisms, and GBA-Mixup as the innovative data augmentation techniques. These components collectively mitigate key limitations in DTA prediction, providing scalable solutions for computational drug discovery.

## Supplementary Material

btaf238_Supplementary_Data

## Data Availability

All datasets utilized in this study are publicly available, and the source code is accessible online at https://github.com/rokieplayer20/MixingDTA.
