# Supplementary material for: MixingDTA: improved drug–target affinity prediction by extending mixup with guilt-by-association
Source: Bioinformatics. 2025 Jul 15;41(Suppl 1):i105–14. doi: 10.1093/bioinformatics/btaf238 (PMC12261493; doi:10.1093/bioinformatics/btaf238)
Supplement: btaf238_Supplementary_Data [file btaf238_supplementary_data.pdf]

# SUPPLEMENTARY INFORMATION FOR: MixingDTA: Improved Drug-Target Affinity Prediction by Extending Mixup with Guilt-By-Association

**Youngoh Kim[1,†], Dongmin Bang[2,3,†], Bonil Koo[2,3], Jungseob Yi[4], Changyun Cho[2,3], Jeonguk Choi[4], and Sun Kim[2,3,4,5,*]**

[1]Bio-MAX Institute, Seoul National University, Seoul, Republic of Korea
[2]Interdisciplinary Program in Bioinformatics, Seoul National University, Seoul, Republic of Korea
[3]AIGENDRUG Co., Ltd., Seoul, Republic of Korea
[4]Interdisciplinary Program in Artificial Intelligence, Seoul National University, Seoul, Republic of Korea
[5]Department of Computer Science and Engineering, Seoul National University, Seoul, Republic of Korea
[†]These authors contributed equally to this work.
[*]For whom the correspondence should be: sunkim.bioinfo@snu.ac.kr

## Contents

**1 Supplementary Figures** **2**
1.1 Supplementary Figure 1. Distribution of labels in DTA datasets . . . 2
1.2 Supplementary Figure 2. Scatter plots for the distances from the ground truth . . . 3
1.3 Supplementary Figure 3. Performance comparison of the integration . . . 4
1.4 Supplementary Figure 4. Performance comparison of the integration ordering . . . 5
1.5 Supplementary Figure 5. Comparison of memory usage . . . 6
1.6 Supplementary Figure 6. Further explanations and KIBA case study . . . 7
1.7 Supplementary Figure 7. Visualization of original and synthesized D-T pairs using PCA . . . 8
**2 Supplementary Tables** **9**
2.1 Supplementary Table 1. Statistics on the string lengths of the datasets . . . 9
2.2 Supplementary Table 2. Selected Hyperparameters of MixingDTA . . . 10
2.3 Supplementary Table 3. Full performance on DAVIS dataset - warm start . . . 11
2.4 Supplementary Table 4. Full performance on KIBA dataset - warm start . . . 12
2.5 Supplementary Table 5. Ablation study of the different perspectives for Multi-view integration on DAVIS dataset . . . 13
2.6 Supplementary Table 6. Ablation study of the different perspectives for Multi-view integration on KIBA dataset . . . 14
2.7 Supplementary Table 7. The performances on GBA-Mixup Case by Case in DAVIS dataset . . . 15
2.8 Supplementary Table 8. The performances on GBA-Mixup Case by Case in KIBA dataset . . . 16
2.9 Supplementary Table 9. Full performance on BindingDB Kd dataset - warm start . . . 17
2.10 Supplementary Table 10. Full performance on PDBbind Refined dataset - warm start . . . 18
2.11 Supplementary Table 11. Cold-start performances of Model-agnosticity Experiment with GBA-Mixup . . . 19
2.12 Supplementary Table 12. Entries for GBA-Mixup Interpolation Between Drugs and Targets Case Study . . . 20
2.13 Supplementary Table 13. The details of interpolation case study for DAVIS . . . 21
2.14 Supplementary Table 14. PDB Entries Utilized for Zero-shot Binding Site Identification . . . 22
2.15 Supplementary Table 15. Efficiency and Accuracy of MEETA's Attention-Free Aggregation . . . 23
**3 Supplementary Methods** **24**
3.1 Comparison model settings . . . 24
3.2 Evaluation Metrics . . . 24
3.3 Case Study on GBA-Mixup Interpolation Between Drugs and Targets . . . 24
3.4 Related Work . . . 25
Pre-trained language models for biomolecules • Drug-Target Affinity Prediction Models • Data Augmentation Through Mixup
3.5 GBA-Mixup Model-agnosticity Experiment Setting . . . 26
**References** **26**

# 1 Supplementary Figures

## 1.1 Supplementary Figure 1. Distribution of labels in DTA datasets

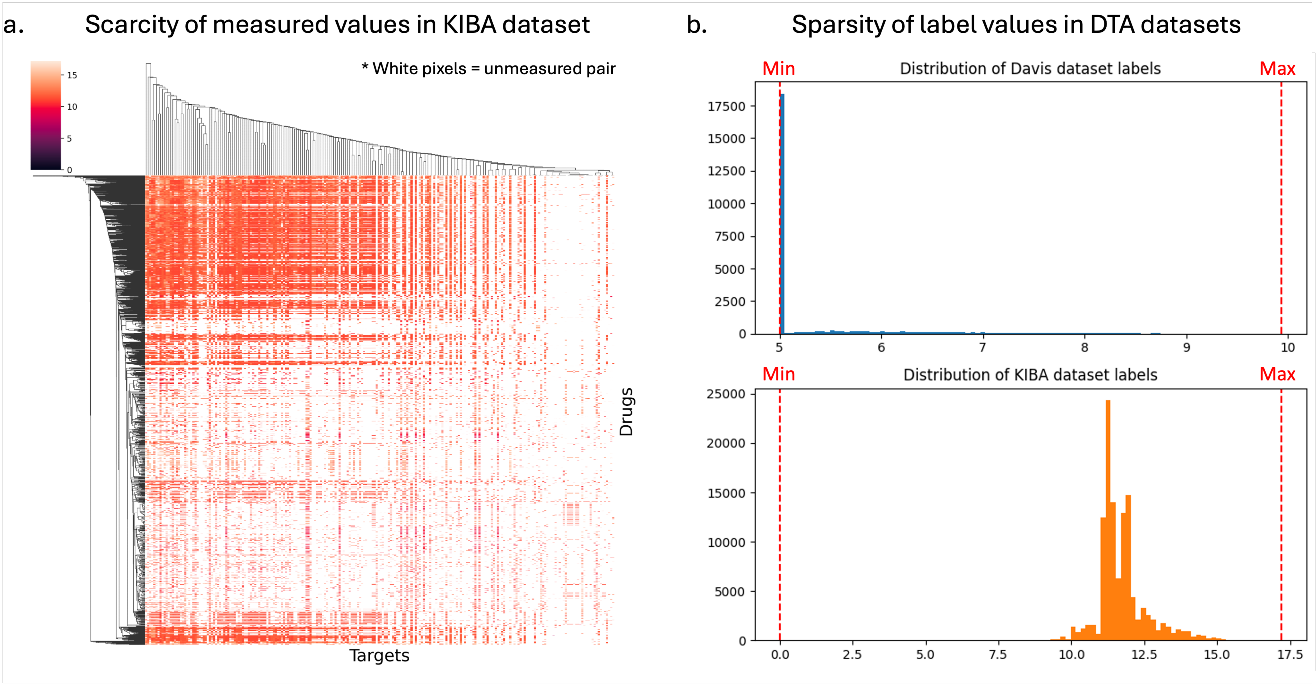


(a) Scarcity of measured values in KIBA dataset. White pixels represent unmeasured points among possible drug-targets pairs. (b) Sparsity of label values in DTA datasets. Among the whole label spectrum, majority of the values are focused in specific range, leaving sparse regions between the minimum and maximum values.

## 1.2 Supplementary Figure 2. Scatter plots for the distances from the ground truth

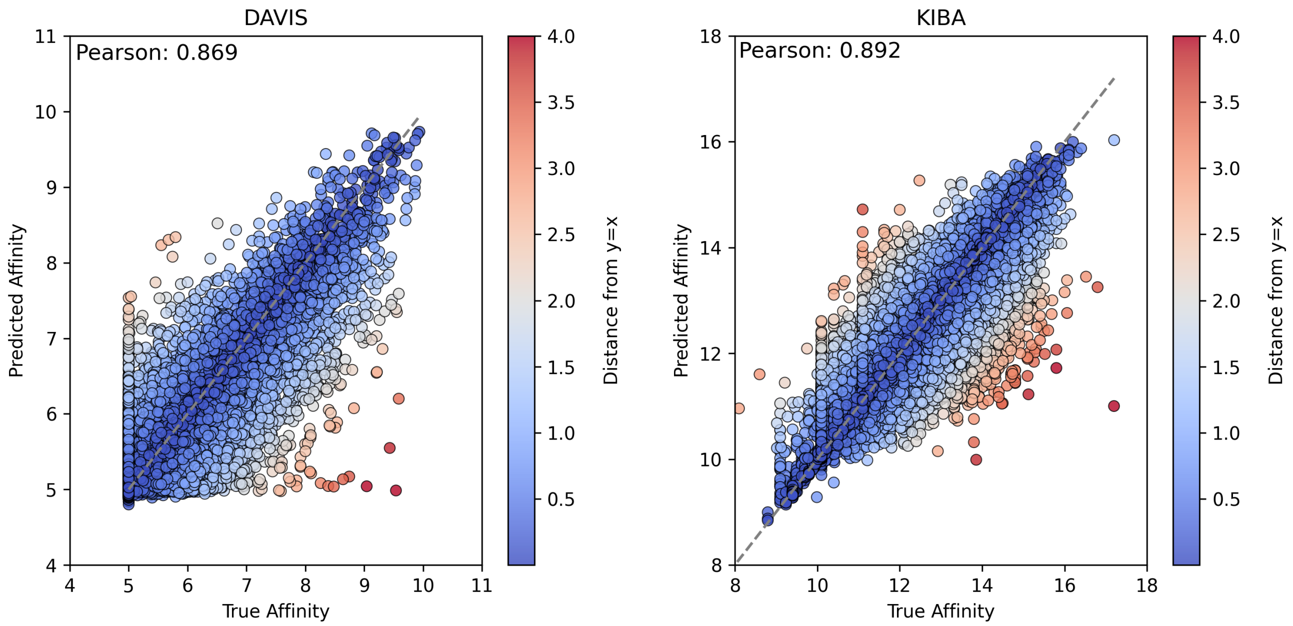


Scatter plots of the predicted values versus ground truths on the DAVIS and KIBA datasets. The distance of each sample from the line $y = x$ is represented using color. As shown, KIBA dataset exhibits the higher Pearson correlation coefficient compared to DAVIS dataset.

### 1.3 Supplementary Figure 3. Performance comparison of the integration

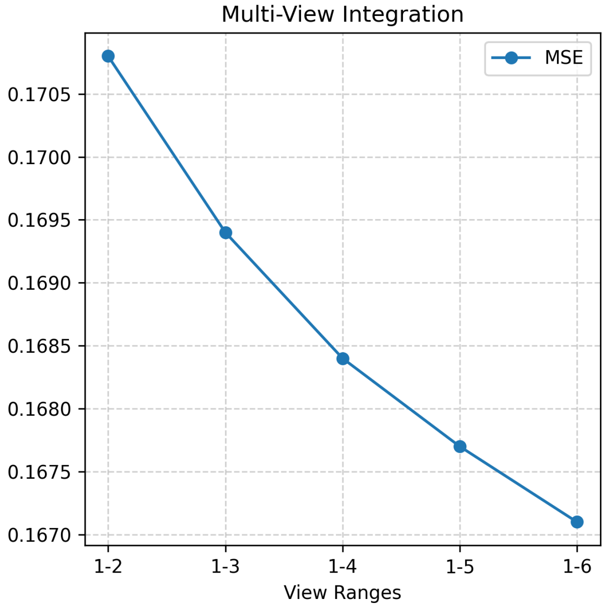


As described in Section 3.1 of this paper, "DT Mixup Pair Sampling," there are six cases. Starting with the first network scenario, additional scenarios were incrementally added and visualized as a graph. The results show performance improvement based on MSE. The experiments were conducted using the DAVIS dataset with random split setting.

We created all possible connection scenarios between D-T nodes based on their components. Among them, we provide additional explanations for two cases:

1. The "None" case: There are no connections. This applies to all existing models in the DTA field. Such a graph is an edgeless graph.

2. The "Complement" case: There are no common components. It represents a network of potential cases. Once the connection scenarios are defined, the GBA principle is practically applied using the C-Mixup method. This assigns higher sampling probabilities to D-T nodes with similar affinity labels.

**The six scenarios assume all possible cases, contributing to the diversification of Mixup. As shown in the figure above, this leads to performance improvement.**

The specific numerical values are as follows: (0.1708, 0.1694, 0.1684, 0.1677, 0.1671).

### 1.4 Supplementary Figure 4. Performance comparison of the integration ordering

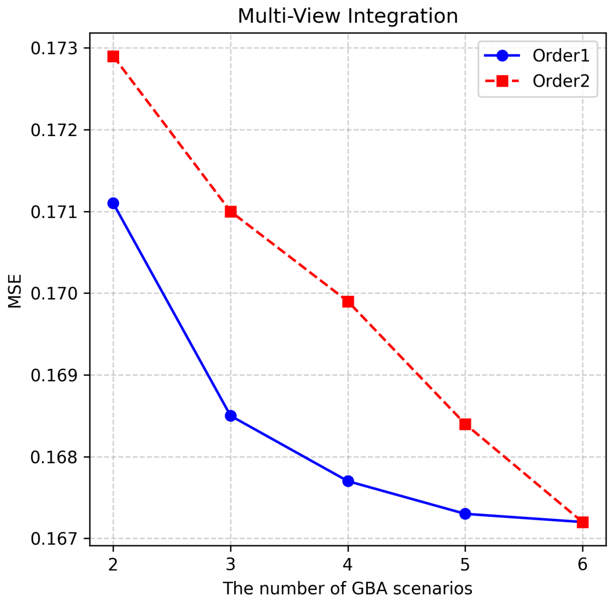


We aim to compare the inclusion order of GBA scenarios in Multi-View Integration from the perspective of MSE performance improvement. Based on the **Ablation Study of the Different Perspectives for Multi-View Integration on the DAVIS Dataset** in the Supplementary Tables, we list the numerical contributions to MSE performance.
None: 0.0012; Protein: 0.0006; Complementary: 0.0005; Protein or Drug: 0.0003; Drug: 0.0001; All-pair: 0.0001.
Order1 prioritizes the inclusion of components with higher contributions in the listed order. Although Drug and All-pair have equal contributions, Drug was included first. Order2 is arranged in the opposite order of Order1. The presented figure shows that Order1 improves MSE performance more quickly than Order2. Therefore, each GBA scenario captures various D-T node connections and contributes differently to improving MixingDTA's performance.

### 1.5 Supplementary Figure 5. Comparison of memory usage

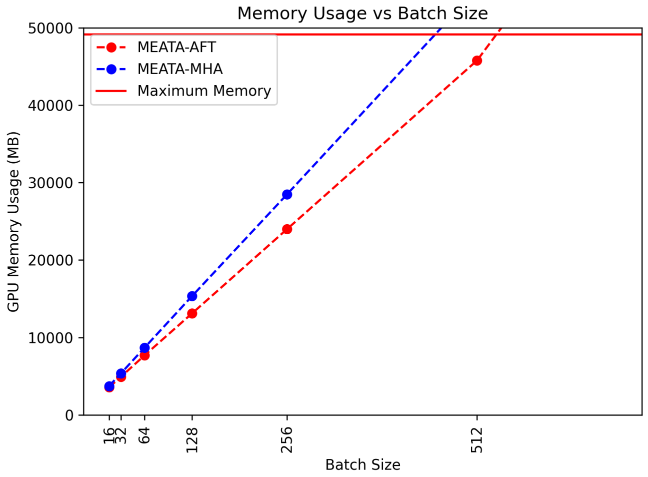


Memory usage of MEETA-MHA and MEETA-AFA in increasing batch sizes. Both models have the same number of trainable parameters (3.8M). Tested on NVIDIA RTX A6000 machine (Maximum 49140 MB).

## 1.6 Supplementary Figure 6. Further explanations and KIBA case study

**a** **Case 1: Interpolation of Drugs by Common Protein (KIBA)**

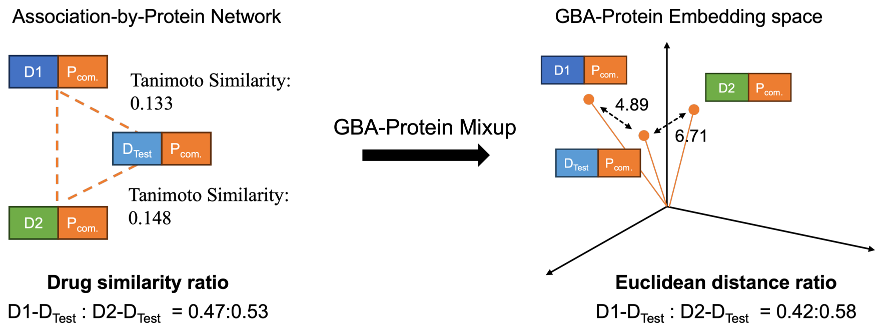


**Binding Affinity: Ground Truth vs Prediction** (Absolute Error)

| D-T pair | Ground Truth | GBA-Protein | No Mixup |
|---|---|---|---|
| **D1-P$_{\mathbf{common}}$** (Train) | 11.10 | 11.09 (0.01) | 11.11 (0.01) |
| **D2-P$_{\mathbf{common}}$** (Train) | 11.90 | 11.96 (0.06) | 11.90 (0.00) |
| **D$_{\mathbf{Test}}$-P$_{\mathbf{common}}$** (Test) | 11.50 | 11.40 (0.10) | 11.24 (0.26) |

**b** **Case 2: Interpolation of Proteins by Common Drug (KIBA)**

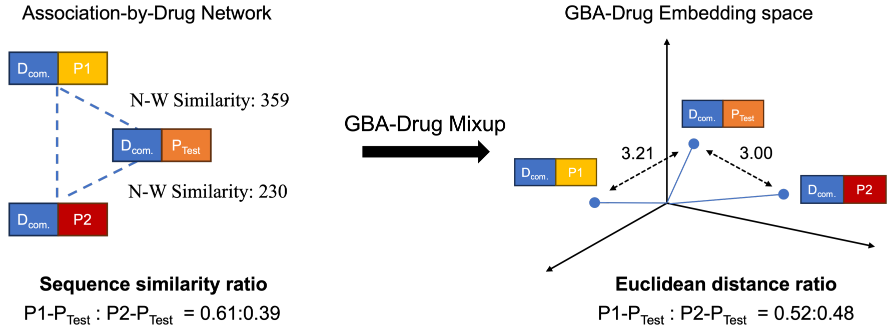


**Binding Affinity: Ground Truth vs Prediction** (Absolute Error)

| D-T pair | Ground Truth | GBA-Drug | No Mixup |
|---|---|---|---|
| **D$_{\mathbf{common}}$-P1** (Train) | 11.10 | 11.12 (0.02) | 11.11 (0.01) |
| **D$_{\mathbf{common}}$-P2** (Train) | 11.80 | 11.85 (0.05) | 11.79 (0.01) |
| **D$_{\mathbf{common}}$-P$_{\mathbf{Test}}$** (Test) | 11.50 | 11.54 (0.04) | 11.89 (0.39) |

This case study was conducted on the KIBA dataset using the same approach as the ‘Case Study on GBA-Mixup Interpolation’ in the main text. The object IDs listed here are included in one of the Supplementary Table 12, titled **Entries for GBA-Mixup Interpolation**.

The effectiveness of the C-Mixup method has already been demonstrated beyond the vision domain, including the DeepDTA model[1]. We fixed the mixing point of the two data points immediately after the Max Pooling layer. In other words, there are two vectors corresponding to max-pooled D-T pairs, and we compute the interpolation point between them in the high-dimensional space. Our approach aims to demonstrate that the interpolated point between two D-T pair vectors is similar to test data that does not exist in the training dataset.

The case studies that visualize these continuous real-valued interpolation points aligns with the characteristics of C-Mixup, which is designed for regression tasks involving real numbers. Therefore, we illustrate the scenario where, if all proteins remain the same, the interpolation between the drugs in the training dataset can generate numerically similar synthetic data to the drugs in the test dataset. Likewise, the same applies when all drugs are identical.

## 1.7 Supplementary Figure 7. Visualization of original and synthesized D-T pairs using PCA

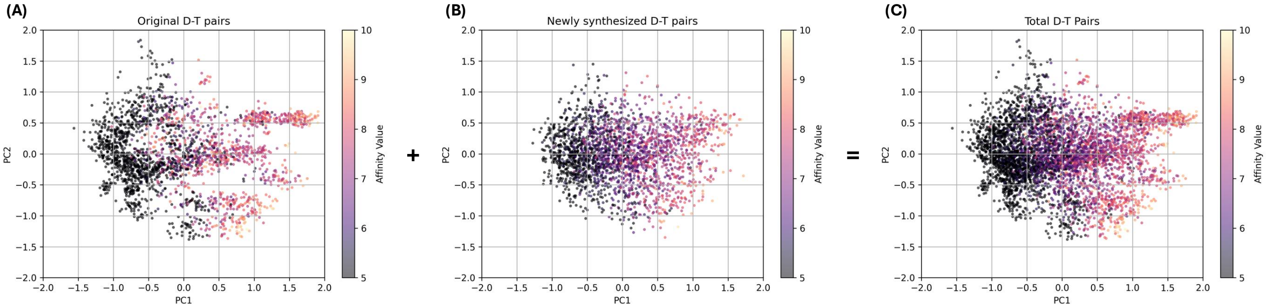


We visualized PCA plots of D-T pair embeddings after max-pooling in the MEETA model, which was trained under the None scenario; (A) Inference on drug-target pairs from the DAVIS dataset was conducted using the MEETA model without Mixup; (B) Mixup is performed once for every drug-target pair in (A) according to the All-Pair scenario; (C) All drug-target pairs from (A) and (B) are combined.

Affinity values in the DAVIS dataset are heavily concentrated between 5.00 and 7.19. This range contains 94.30 % of the total values within the overall range of [5.00, 9.94]. We perform PCA on the DAVIS dataset to investigate how GBA-Mixup addresses sparsity outside the [5.00, 7.19] range. The dense range is [5.00, 7.19], and the sparse range covers all remaining values. For clearer visual analysis, only 10 % of data points from the dense range were used in the PCA. In plot (A), the points are dispersed, showing empty spaces between clusters. In plot (B), Mixup was applied once to each point according to the All-pair scenario. As affinity values are mixed, they fill the empty spaces towards the center. When all points are combined as shown in plots (C), it becomes clear that the overall sparsity in the embedding space has been resolved.

# 2 Supplementary Tables

## 2.1 Supplementary Table 1. Statistics on the string lengths of the datasets

Statics of character lengths and total number of samples in benchmark datasets

| **Datasets** | **Drug** | | | | **Protein** | | | |
|---|---|---|---|---|---|---|---|---|
| | Minimum | Average | Maximum | Total # | Minimum | Average | Maximum | Total # |
| DAVIS | 32 | 54.2 | 81 | 68 | 244 | 744.9 | 2549 | 379 |
| KIBA | 14 | 46.8 | 532 | 2,068 | 215 | 730.6 | 4128 | 229 |

## 2.2 Supplementary Table 2. Selected Hyperparameters of MixingDTA

The experimental settings used for training MixingDTA are as follows. The $\sigma$ of KDE (Kernel Density Estimation) represents the bandwidth of the kernel required to calculate the sampling probability. $\alpha$ is used to sample the interpolation factor $\lambda$ from the Beta distribution Beta$(\alpha, \alpha)$. In other model-agnostic experiments, the training strategies from each model's original paper are followed. The hyperparameters for mixup and step 2 remain consistent across all experiments.

| **Hyperparameters** | **MixingDTA** | **Model-agnostic training (DeepDTA)** |
|---|---|---|
| Base learning rate | 5e-5 | 5e-5 |
| Max learning rate | 5e-4 | n/a |
| Weight decay | 1e-4 | n/a |
| Dropout rate | 0.15 | 0.1 |
| Batch size | 64 | 64 |
| Optimizer | AdamW | Adam |
| Scheduler | CyclicLR | n/a |
| Scheduler mode | triangular2 | n/a |
| Max epoch | 500 | 500 |
| Patience | 30 | 30 |
| KDE type | Gaussian | Gaussian |
| KDE bandwidth ($\sigma$) | 21 | 21 |
| $\alpha$ in Beta distribution | 2.0 | 2.0 |

## 2.3 Supplementary Table 3. Full performance on DAVIS dataset - warm start

| | MSE (std) | RMSE (std) | MAE (std) | R2 (std) | CI (std) | $r_m^2$ (std) | Pearson (std) | Spearman (std) | AUPR (std) |
|---|---|---|---|---|---|---|---|---|---|
| CSCo-DTA | 0.283 (0.0177) | 0.532 (0.0167) | 0.319 (0.0104) | 0.584 (0.0163) | 0.862 (0.0050) | 0.559 (0.0171) | 0.766 (0.0109) | 0.641 (0.0074) | 0.605 (0.0235) |
| MD-CT-DTA | 0.275 (0.0072) | 0.524 (0.0068) | 0.308 (0.0045) | 0.596 (0.0117) | 0.857 (0.0061) | 0.555 (0.0244) | 0.778 (0.0067) | 0.631 (0.0092) | 0.623 (0.0147) |
| DeepDTA | 0.243 (0.0140) | 0.493 (0.0141) | 0.263 (0.0074) | 0.643 (0.0140) | 0.865 (0.0088) | 0.614 (0.0219) | 0.806 (0.0069) | 0.642 (0.0120) | 0.653 (0.0152) |
| GraphDTA | 0.241 (0.0106) | 0.491 (0.0108) | 0.280 (0.0078) | 0.645 (0.0146) | 0.869 (0.0076) | 0.632 (0.0237) | 0.804 (0.0082) | 0.650 (0.0114) | 0.667 (0.0124) |
| ML-DTI | 0.232 (0.0092) | 0.481 (0.0096) | 0.249 (0.0111) | 0.660 (0.0206) | 0.873 (0.0064) | 0.635 (0.0330) | 0.815 (0.0117) | 0.654 (0.0088) | 0.681 (0.0265) |
| MGraphDTA | 0.217 (0.0150) | 0.465 (0.0164) | 0.236 (0.0057) | 0.681 (0.0228) | 0.879 (0.0075) | 0.673 (0.0235) | 0.826 (0.0134) | 0.664 (0.0102) | 0.676 (0.0148) |
| AttentionDTA | 0.215 (0.0090) | 0.463 (0.0096) | 0.226 (0.0065) | 0.684 (0.0159) | 0.879 (0.0066) | 0.663 (0.0284) | 0.829 (0.0086) | 0.663 (0.0085) | 0.672 (0.0084) |
| MEETA | 0.181 (0.0096) | 0.425 (0.0112) | 0.218 (0.0005) | 0.734 (0.0103) | 0.889 (0.0099) | 0.711 (0.0217) | 0.860 (0.0062) | 0.677 (0.0149) | 0.716 (0.0138) |
| MixingDTA | 0.167 (0.0095) | 0.408 (0.0115) | 0.219 (0.0053) | 0.755 (0.0133) | 0.906 (0.0046) | 0.745 (0.0165) | 0.869 (0.0075) | 0.703 (0.0061) | 0.730 (0.0152) |

## 2.4 Supplementary Table 4. Full performance on KIBA dataset - warm start

| | MSE (std) | RMSE (std) | MAE (std) | R2 (std) | CI (std) | $r_m^2$ (std) | Pearson (std) | Spearman (std) | AUPR (std) |
|---|---|---|---|---|---|---|---|---|---|
| MD-CT-DTA | 0.237 (0.0162) | 0.487 (0.0166) | 0.312 (0.0138) | 0.658 (0.0215) | 0.825 (0.0089) | 0.626 (0.0294) | 0.814 (0.0146) | 0.791 (0.0152) | 0.721 (0.0161) |
| CSCo-DTA | 0.203 (0.0131) | 0.451 (0.0143) | 0.276 (0.0100) | 0.706 (0.0174) | 0.852 (0.0052) | 0.698 (0.0159) | 0.841 (0.0105) | 0.831 (0.0078) | 0.745 (0.0107) |
| DeepDTA | 0.190 (0.0053) | 0.436 (0.0061) | 0.247 (0.0057) | 0.725 (0.0053) | 0.865 (0.0031) | 0.691 (0.0083) | 0.854 (0.0038) | 0.846 (0.0052) | 0.772 (0.0069) |
| ML-DTI | 0.186 (0.0040) | 0.431 (0.0046) | 0.245 (0.0034) | 0.733 (0.0070) | 0.868 (0.0032) | 0.706 (0.0100) | 0.857 (0.0044) | 0.848 (0.0050) | 0.774 (0.0072) |
| GraphDTA | 0.177 (0.0033) | 0.420 (0.0039) | 0.247 (0.0060) | 0.745 (0.0035) | 0.868 (0.0025) | 0.733 (0.0072) | 0.863 (0.0020) | 0.852 (0.0031) | 0.779 (0.0050) |
| AttentionDTA | 0.167 (0.0027) | 0.409 (0.0033) | 0.224 (0.0026) | 0.759 (0.0049) | 0.880 (0.0017) | 0.732 (0.0116) | 0.872 (0.0027) | 0.865 (0.0031) | 0.788 (0.0032) |
| MGraphDTA | 0.148 (0.0029) | 0.384 (0.0037) | 0.200 (0.0042) | 0.787 (0.0051) | 0.894 (0.0015) | 0.775 (0.0103) | 0.887 (0.0029) | 0.881 (0.0024) | 0.807 (0.0026) |
| MEETA | 0.153 (0.0019) | 0.391 (0.0025) | 0.215 (0.0023) | 0.780 (0.0047) | 0.881 (0.0028) | 0.768 (0.0150) | 0.883 (0.0025) | 0.868 (0.0040) | 0.792 (0.0056) |
| MixingDTA | 0.142 (0.0013) | 0.376 (0.0018) | 0.221 (0.0024) | 0.795 (0.0005) | 0.880 (0.0017) | 0.780 (0.0129) | 0.892 (0.0004) | 0.876 (0.0031) | 0.798 (0.0023) |

## 2.5 Supplementary Table 5. Ablation study of the different perspectives for Multi-view integration on DAVIS dataset

| | MSE (std) | RMSE (std) | MAE (std) | R2 (std) | CI (std) | $r_m^2$ (std) | Pearson (std) | Spearman (std) | AUPR (std) |
|---|---|---|---|---|---|---|---|---|---|
| w/o None | 0.168 (0.0094) | 0.410 (0.0115) | 0.221 (0.0051) | 0.753 (0.0132) | 0.906 (0.0044) | 0.746 (0.0150) | 0.868 (0.0074) | 0.704 (0.0057) | 0.728 (0.0161) |
| w/o All-pair | 0.167 (0.0092) | 0.409 (0.0111) | 0.218 (0.0062) | 0.755 (0.0130) | 0.906 (0.0042) | 0.745 (0.0177) | 0.869 (0.0074) | 0.704 (0.0055) | 0.728 (0.0128) |
| w/o Drug | 0.167 (0.0088) | 0.409 (0.0107) | 0.218 (0.0050) | 0.755 (0.0125) | 0.906 (0.0045) | 0.743 (0.0204) | 0.869 (0.0069) | 0.702 (0.0057) | 0.732 (0.0112) |
| w/o Protein | 0.168 (0.0099) | 0.410 (0.0120) | 0.219 (0.0062) | 0.754 (0.0136) | 0.906 (0.0055) | 0.747 (0.0148) | 0.869 (0.0078) | 0.703 (0.0075) | 0.729 (0.0141) |
| w/o Protein or Drug | 0.168 (0.0095) | 0.409 (0.0116) | 0.220 (0.0035) | 0.755 (0.0132) | 0.906 (0.0049) | 0.741 (0.0118) | 0.869 (0.0074) | 0.703 (0.0063) | 0.728 (0.0112) |
| w/o Complement | 0.168 (0.0093) | 0.409 (0.0112) | 0.220 (0.0054) | 0.754 (0.0134) | 0.906 (0.0047) | 0.741 (0.0217) | 0.869 (0.0076) | 0.703 (0.0063) | 0.734 (0.0147) |
| MixingDTA | 0.167 (0.0095) | 0.408 (0.0115) | 0.219 (0.0053) | 0.755 (0.0133) | 0.906 (0.0046) | 0.745 (0.0165) | 0.869 (0.0075) | 0.703 (0.0061) | 0.730 (0.0152) |

## 2.6 Supplementary Table 6. Ablation study of the different perspectives for Multi-view integration on KIBA dataset

The removal of one scenario in training step2 has the minimal impact. However, the tables of "The Performances on GBA-Mixup Case by Case" highlight the powerful effect of multi-view integration.

| | MSE (std) | RMSE (std) | MAE (std) | R2 (std) | CI (std) | $r_m^2$ (std) | Pearson (std) | Spearman (std) | AUPR (std) |
|---|---|---|---|---|---|---|---|---|---|
| w/o None | 0.143 (0.0015) | 0.378 (0.0020) | 0.224 (0.0026) | 0.795 (0.0004) | 0.879 (0.0016) | 0.780 (0.0070) | 0.892 (0.0003) | 0.875 (0.0030) | 0.799 (0.0028) |
| w/o All-pair | 0.142 (0.0015) | 0.377 (0.0020) | 0.223 (0.0020) | 0.796 (0.0003) | 0.880 (0.0015) | 0.785 (0.0101) | 0.892 (0.0002) | 0.876 (0.0030) | 0.798 (0.0021) |
| w/o Drug | 0.143 (0.0013) | 0.378 (0.0017) | 0.223 (0.0020) | 0.795 (0.0004) | 0.879 (0.0014) | 0.781 (0.0090) | 0.892 (0.0003) | 0.875 (0.0029) | 0.798 (0.0029) |
| w/o Protein | 0.143 (0.0014) | 0.378 (0.0018) | 0.222 (0.0022) | 0.795 (0.0013) | 0.880 (0.0018) | 0.783 (0.0047) | 0.892 (0.0008) | 0.876 (0.0032) | 0.798 (0.0035) |
| w/o Protein or Drug | 0.143 (0.0012) | 0.378 (0.0015) | 0.223 (0.0022) | 0.795 (0.0011) | 0.879 (0.0017) | 0.784 (0.0084) | 0.892 (0.0007) | 0.875 (0.0030) | 0.798 (0.0029) |
| w/o Complement | 0.143 (0.0013) | 0.378 (0.0017) | 0.222 (0.0022) | 0.795 (0.0011) | 0.880 (0.0018) | 0.783 (0.0122) | 0.892 (0.0005) | 0.876 (0.0033) | 0.798 (0.0032) |
| MixingDTA | 0.142 (0.0013) | 0.376 (0.0018) | 0.221 (0.0024) | 0.795 (0.0005) | 0.880 (0.0017) | 0.780 (0.0129) | 0.892 (0.0004) | 0.876 (0.0031) | 0.798 (0.0023) |

## 2.7 Supplementary Table 7. The performances on GBA-Mixup Case by Case in DAVIS dataset

| | MSE (std) | RMSE (std) | MAE (std) | R2 (std) | CI (std) | $r_m^2$ (std) | Pearson (std) | Spearman (std) | AUPR (std) |
|---|---|---|---|---|---|---|---|---|---|
| None | 0.181 (0.0096) | 0.425 (0.0112) | 0.219 (0.0005) | 0.735 (0.0103) | 0.889 (0.0099) | 0.711 (0.0217) | 0.860 (0.0062) | 0.677 (0.0149) | 0.716 (0.0138) |
| All-pair | 0.180 (0.0096) | 0.424 (0.0112) | 0.234 (0.0067) | 0.736 (0.0124) | 0.896 (0.0059) | 0.730 (0.0118) | 0.859 (0.0074) | 0.688 (0.0080) | 0.719 (0.0170) |
| Drug | 0.181 (0.0093) | 0.426 (0.0109) | 0.231 (0.0031) | 0.734 (0.0105) | 0.897 (0.0041) | 0.727 (0.0187) | 0.858 (0.0070) | 0.690 (0.0049) | 0.717 (0.0097) |
| Protein | 0.181 (0.0050) | 0.425 (0.0059) | 0.233 (0.0018) | 0.735 (0.0099) | 0.895 (0.0024) | 0.725 (0.0176) | 0.858 (0.0064) | 0.687 (0.0011) | 0.717 (0.0100) |
| Protein or Drug | 0.184 (0.0071) | 0.429 (0.0082) | 0.239 (0.0059) | 0.730 (0.0128) | 0.897 (0.0042) | 0.721 (0.0214) | 0.856 (0.0077) | 0.689 (0.0058) | 0.715 (0.0176) |
| Complement | 0.181 (0.0100) | 0.426 (0.0117) | 0.239 (0.0110) | 0.734 (0.0144) | 0.896 (0.0040) | 0.720 (0.0248) | 0.857 (0.0082) | 0.689 (0.0044) | 0.724 (0.0162) |
| MixingDTA | 0.167 (0.0095) | 0.408 (0.0115) | 0.219 (0.0053) | 0.755 (0.0133) | 0.906 (0.0046) | 0.745 (0.0165) | 0.869 (0.0075) | 0.703 (0.0061) | 0.730 (0.0152) |

## 2.8 Supplementary Table 8. The performances on GBA-Mixup Case by Case in KIBA dataset

Even though the performance of individual cases does not surpass that of the None case (MEETA), combining all perspectives into one reveals a synergistic effect that enhances overall performance.

| | MSE (std) | RMSE (std) | MAE (std) | R2 (std) | CI (std) | $r_m^2$ (std) | Pearson (std) | Spearman (std) | AUPR (std) |
|---|---|---|---|---|---|---|---|---|---|
| None | 0.153 (0.0019) | 0.391 (0.0025) | 0.215 (0.0023) | 0.780 (0.0047) | 0.882 (0.0028) | 0.768 (0.0150) | 0.884 (0.0025) | 0.869 (0.0040) | 0.792 (0.0056) |
| All-pair | 0.156 (0.0025) | 0.395 (0.0031) | 0.243 (0.0034) | 0.776 (0.0015) | 0.866 (0.0019) | 0.764 (0.0089) | 0.881 (0.0009) | 0.857 (0.0027) | 0.783 (0.0040) |
| Drug | 0.154 (0.0032) | 0.392 (0.0041) | 0.230 (0.0039) | 0.779 (0.0053) | 0.874 (0.0037) | 0.759 (0.0083) | 0.883 (0.0032) | 0.864 (0.0059) | 0.787 (0.0050) |
| Protein | 0.155 (0.0017) | 0.393 (0.0021) | 0.239 (0.0031) | 0.778 (0.0014) | 0.868 (0.0021) | 0.766 (0.0126) | 0.882 (0.0007) | 0.859 (0.0037) | 0.784 (0.0042) |
| Protein or Drug | 0.154 (0.0036) | 0.392 (0.0046) | 0.238 (0.0062) | 0.779 (0.0045) | 0.869 (0.0041) | 0.766 (0.0078) | 0.883 (0.0026) | 0.860 (0.0050) | 0.783 (0.0047) |
| Complement | 0.156 (0.0018) | 0.394 (0.0023) | 0.244 (0.0026) | 0.777 (0.0033) | 0.865 (0.0021) | 0.762 (0.0075) | 0.881 (0.0019) | 0.856 (0.0031) | 0.783 (0.0040) |
| MixingDTA | 0.142 (0.0013) | 0.376 (0.0018) | 0.221 (0.0024) | 0.795 (0.0005) | 0.880 (0.0017) | 0.780 (0.0129) | 0.892 (0.0004) | 0.876 (0.0031) | 0.798 (0.0023) |

## 2.9 Supplementary Table 9. Full performance on BindingDB Kd dataset - warm start

BindingDB Kd[2,3] is also available from the TDC database, similar to DAVIS and KIBA. D-T pairs with mismatched string and ID mappings were removed.

| | MSE (std) | RMSE (std) | MAE (std) | R2 (std) | CI (std) | $r_m^2$ (std) | Pearson (std) | Spearman (std) |
|---|---|---|---|---|---|---|---|---|
| MD-CT-DTA | 0.564 (0.0176) | 0.751 (0.0116) | 0.470 (0.0061) | 0.693 (0.0115) | 0.848 (0.0013) | 0.640 (0.0138) | 0.836 (0.0067) | 0.765 (0.0029) |
| DeepDTA | 0.525 (0.0121) | 0.724 (0.0084) | 0.433 (0.0060) | 0.714 (0.0110) | 0.856 (0.0027) | 0.669 (0.0135) | 0.849 (0.0056) | 0.779 (0.0063) |
| MGraphDTA | 0.497 (0.0153) | 0.705 (0.0109) | 0.392 (0.0069) | 0.730 (0.0088) | 0.864 (0.0023) | 0.681 (0.0181) | 0.857 (0.0044) | 0.790 (0.0041) |
| ML-DTI | 0.496 (0.0152) | 0.704 (0.0108) | 0.406 (0.0085) | 0.730 (0.0084) | 0.860 (0.0070) | 0.694 (0.0282) | 0.857 (0.0047) | 0.783 (0.0116) |
| GraphDTA | 0.495 (0.0100) | 0.704 (0.0071) | 0.419 (0.0046) | 0.731 (0.0070) | 0.860 (0.0025) | 0.692 (0.0160) | 0.856 (0.0040) | 0.786 (0.0058) |
| AttentionDTA | 0.462 (0.0074) | 0.680 (0.0054) | 0.381 (0.0036) | 0.748 (0.0073) | 0.869 (0.0033) | 0.702 (0.0125) | 0.867 (0.0040) | 0.797 (0.0069) |
| MEETA | 0.414 (0.0103) | 0.643 (0.0081) | 0.367 (0.0042) | 0.775 (0.0078) | 0.874 (0.0042) | 0.754 (0.0144) | 0.881 (0.0044) | 0.807 (0.0080) |
| MixingDTA | **0.383 (0.0082)** | **0.619 (0.0066)** | **0.366 (0.0027)** | **0.792 (0.0056)** | **0.883 (0.0043)** | **0.782 (0.0146)** | **0.890 (0.0030)** | **0.823 (0.0076)** |

## 2.10 Supplementary Table 10. Full performance on PDBbind Refined dataset - warm start

| | MSE (std) | RMSE (std) | MAE (std) | R2 (std) | CI (std) | $r_m^2$ (std) | Pearson (std) | Spearman (std) |
|---|---|---|---|---|---|---|---|---|
| MD-CT-DTA | 2.404 (0.1001) | 1.550 (0.0326) | 1.189 (0.0342) | 0.405 (0.0214) | 0.737 (0.0098) | 0.374 (0.0246) | 0.662 (0.0161) | 0.653 (0.0230) |
| GraphDTA | 2.273 (0.1028) | 1.507 (0.0344) | 1.159 (0.0439) | 0.437 (0.0187) | 0.743 (0.0084) | 0.399 (0.0180) | 0.677 (0.0116) | 0.666 (0.0193) |
| MGraphDTA | 2.234 (0.0972) | 1.494 (0.0330) | 1.116 (0.0244) | 0.446 (0.0358) | 0.751 (0.0088) | 0.411 (0.0294) | 0.688 (0.0202) | 0.679 (0.0227) |
| DeepDTA | 2.069 (0.1741) | 1.437 (0.0604) | 1.099 (0.0491) | 0.488 (0.0342) | 0.767 (0.0059) | 0.449 (0.0181) | 0.724 (0.0128) | 0.722 (0.0140) |
| ML-DTI | 1.933 (0.0965) | 1.390 (0.0348) | 1.075 (0.0301) | 0.521 (0.0180) | 0.769 (0.0043) | 0.493 (0.0280) | 0.731 (0.0099) | 0.729 (0.0075) |
| AttentionDTA | 1.858 (0.1038) | 1.363 (0.0381) | 1.049 (0.0311) | 0.540 (0.0134) | 0.774 (0.0054) | 0.525 (0.0346) | 0.743 (0.0102) | 0.738 (0.0137) |
| MEETA | 1.837 (0.1518) | 1.354 (0.0558) | 1.024 (0.0547) | 0.546 (0.0276) | 0.777 (0.0088) | 0.514 (0.0388) | 0.744 (0.0149) | 0.742 (0.0177) |
| MixingDTA | **1.783 (0.1473)** | **1.334 (0.0554)** | **1.008 (0.0485)** | **0.559 (0.0249)** | **0.782 (0.0072)** | **0.529 (0.0294)** | **0.754 (0.0142)** | **0.751 (0.0150)** |

PDBbind Refined[4] was obtained from the following URL[1]. It was then preprocessed for use in the study. To ensure consistency between strings and their corresponding IDs, new IDs were assigned to each unique string. Subsequently, duplicate D-T pairs were removed.

This dataset is significantly smaller compared to the others. Additionally, constructing a protein-based GBA network is challenging. Therefore, we adjusted the training conditions. The modifications are as follows:

Patience: 50, Dropout rate: 0.1, Weight decay: 5e-5, Scheduler mode: triangular.

Protein-related GBA scenarios, including "Protein" and "Protein or Drug," were excluded from multi-view integration.

[1] MahaThafar/Affinity2Vec/blob/main/PDBBind_Refined/All_PDBbind_info.csv. Accessed 18 Jan. 2025.

### 2.11 Supplementary Table 11. Cold-start performances of Model-agnosticity Experiment with GBA-Mixup

Performance on DAVIS datasets with drug- and target-cold start (blind split) setting. Mean and standard deviation of 5-fold cross validation are provided. Best performances in bold and second-best underlined.

| | **DAVIS (Unseen/Cold Target)** | | | | **DAVIS (Unseen/Cold Drug)** | | | |
|---|---|---|---|---|---|---|---|---|
| | MSE (↓) | CI (↑) | $r_m^2$ (↑) | AUPR (↑) | MSE (↓) | CI (↑) | $r_m^2$ (↑) | AUPR (↑) |
| DeepDTA | 0.358 (0.0233) | 0.807 (0.0129) | 0.407 (0.0182) | 0.508 (0.0331) | 0.714 (0.0533) | 0.644 (0.0422) | 0.065 (0.0403) | 0.202 (0.0627) |
| MixingDTA (DeepDTA) | 0.338 (0.0101) | 0.825 (0.0028) | 0.426 (0.0221) | 0.495 (0.0187) | 0.573 (0.0237) | 0.720 (0.0198) | 0.179 (0.0284) | 0.324 (0.0493) |
| AttentionDTA | 0.330 (0.0089) | 0.823 (0.0062) | 0.413 (0.0169) | 0.531 (0.0225) | 0.702 (0.0640) | 0.666 (0.0342) | 0.108 (0.0302) | 0.257 (0.0263) |
| MixingDTA (AttentionDTA) | 0.294 (0.0031) | 0.843 (0.0020) | 0.487 (0.0041) | 0.543 (0.0127) | 0.589 (0.0258) | 0.724 (0.0109) | 0.164 (0.0266) | 0.309 (0.0357) |
| ML-DTI | 0.350 (0.0167) | 0.813 (0.0070) | 0.408 (0.0229) | 0.518 (0.0286) | 0.688 (0.0907) | 0.672 (0.0321) | 0.111 (0.0582) | 0.269 (0.0790) |
| MixingDTA (ML-DTI) | 0.304 (0.0028) | 0.842 (0.0055) | 0.494 (0.0126) | 0.542 (0.0047) | <u>0.561 (0.0376)</u> | 0.733 (0.0161) | 0.201 (0.0492) | 0.316 (0.0635) |
| MEETA | <u>0.244 (0.0099)</u> | <u>0.857 (0.0032)</u> | <u>0.531 (0.0239)</u> | <u>0.631 (0.0131)</u> | 0.573 (0.0288) | <u>0.734 (0.0066)</u> | <u>0.238 (0.0130)</u> | <u>0.519 (0.0181)</u> |
| **MixingDTA** | **0.231 (0.0042)** | **0.874 (0.0017)** | **0.567 (0.0144)** | **0.634 (0.0105)** | **0.538 (0.0123)** | **0.754 (0.0048)** | **0.258 (0.0072)** | **0.523 (0.0110)** |

### 2.12 Supplementary Table 12. Entries for GBA-Mixup Interpolation Between Drugs and Targets Case Study

The following IDs are from the DAVIS dataset.

| Object Name | ID |
|---|---|
| $Drug_1$ | 5291 |
| $Drug_2$ | 126565 |
| $Drug_{test}$ | 25243800 |
| $Protein_{common}$ | MLCK |
| $Protein_1$ | CTK |
| $Protein_2$ | EGFR(T790M) |
| $Protein_{test}$ | ABL1p |
| $Drug_{common}$ | 156414 |

The following IDs are from the KIBA dataset.

| Object Name | ID |
|---|---|
| $Drug_1$ | CHEMBL1972820 |
| $Drug_2$ | CHEMBL1967878 |
| $Drug_{\text{test}}$ | CHEMBL1974310 |
| $Protein_{\text{common}}$ | Q9HBY8 |
| $Protein_1$ | Q9Y243 |
| $Protein_2$ | O75582 |
| $Protein_{\text{test}}$ | O14965 |
| $Drug_{\text{common}}$ | CHEMBL1236126 |

## 2.13 Supplementary Table 13. The details of interpolation case study for DAVIS

| Ratio | $D_1 - D_{test} : D_2 - D_{test}$ |
|---|---|
| Tanimoto similarity | 0.48:0.52 |
| Affinity label | 0.49:0.51 |
| Euclidean distance | 0.50: 0.50 |
| Ratio | $P_1 - P_{test} : P_2 - P_{test}$ |
| N-W similarity | 0.54:0.46 |
| Affinity label | 0.57:0.43 |
| Euclidean distance | 0.50 : 0.50 |

The table shows the distance ratio on the embedding and the affinity label. GBA-Mixup is performed based on these two factors.

## 2.14 Supplementary Table 14. PDB Entries Utilized for Zero-shot Binding Site Identification

PDB identification codes of the imatinib-docked structures utilized for zero-shot binding site identifiation case study. The binding site residues are defined as residues within 4Å of imatinib.

| Drug | **PDB identification codes** |
|---|---|
| Imatinib | 1T46A, 2PL0A, 7N9GA, 2OIQA, 6JOLA, 3OEZA, 3GVUA, 4CSVA, 3FW1A, 3K5VA, 1IEPA, 6NPUA, 4R7IA, 1XBBA, 3MS9A, 2HYYA, 5MQTA, 3HECA, 6KTNA, 4BKJA |

### 2.15 Supplementary Table 15. Efficiency and Accuracy of MEETA's Attention-Free Aggregation

Zero-shot binding site identification performance on Imatinib dataset. Mean and standard deviation of 20 co-crystalized structures are provided, with the best performances in bold.

| | **MCC** | **F1** | **Avg. Prec.** | **AUROC** |
|---|---|---|---|---|
| AttentionDTA | 0.00 (0.056) | 0.06 (0.055) | 0.07 (0.062) | 0.50 (0.027) |
| MEETA-MHA | 0.02 (0.059) | 0.11 (0.038) | 0.08 (0.023) | **0.52 (0.052)** |
| MEETA-AFA | -0.03 (0.063) | 0.10 (0.042) | 0.07 (0.015) | 0.46 (0.070) |
| MixingDTA | **0.06 (0.057)** | **0.14 (0.032)** | **0.10 (0.034)** | 0.49 (0.077) |

# 3 Supplementary Methods

## 3.1 Comparison model settings

**CSCo-DTA**[5] CSCo-DTA levarages drug-target protein bipartite graph. CSCo-DTA is not capable of inferring entities that are not included in the train graph. We utilized the original codes and data from the authors' repository (`https://github.com/23AIBox/23AIBox-CSCo-DTA`).

## 3.2 Evaluation Metrics

In our study, we evaluated the performance of MixingDTA and baseline models using statistical metrics. These include Mean Square Error (MSE), Concordance Index (CI), $\mathrm{r_m}^2$, and Area Under the Precision-Recall (AUPR) score.

MSE measures the error between the true values and the model's predictions. The formula for MSE is expressed as follows:

$$\mathrm{MSE} = \frac{1}{N}\sum_{i=1}^{N}(y_i - \hat{y}_i)^2$$

where $N$ represents the number of DT pairs; $y_i$ and $\hat{y}_i$ correspond to the $i$-th true affinity and prediction, respectively. The concordance index (C-index) is a metric used to evaluate the performance of survival models by comparing predicted scores with actual survival times. It measures the agreement between predicted rankings and observed event times. Specifically, it assesses whether the model correctly predicts that a subject with a longer observed survival time has a higher predicted score than one with a shorter survival time.

The C-Index[6] ranges from 0 to 1. A value of 0.5 indicates random predictions, while 1.0 represents perfect concordance. A value of 0.0 reflects perfect anti-concordance.

The formula for calculating the C-Index is:

$$\text{C-Index} = \frac{\mathrm{N_{correct}} + 0.5 \cdot \mathrm{N_{tied}}}{\mathrm{N_{total}}}$$

Here, $\mathrm{N_{correct}}$ counts the number of correctly ordered pairs (i.e., if $t_x > t_y$, then $s_x > s_y$). $\mathrm{N_{tied}}$ counts pairs with equal predicted scores, and $\mathrm{N_{total}}$ includes all possible pairs that can be evaluated.

$r_m^2$ metric[78] is a measure designed to evaluate a model's external predictive performance. This index plays a role in assessing how well the model explains the relationship between actual and predicted values, particularly in determining its predictive power on test dataset. It is calculated as follows:

$$r_m^2 = r^2\left(1 - \sqrt{r^2 - r_0^2}\right)$$

$r^2$ and $r_0^2$ are both squared correlation coefficients; however, the former includes an intercept, while the latter does not.

The Area Under the Precision-Recall Curve (AUPR) is a key metric for evaluating the recommendation capabilities of the model predictions based on binary labels. We evaluated the models using thresholds of 7 for the DAVIS dataset and 12.1 for the KIBA dataset, respectively, as proposed in a previous study[9].

## 3.3 Case Study on GBA-Mixup Interpolation Between Drugs and Targets

This section describes the methodology used to evaluate the GBA-Mixup approach in interpolating drug–target (D–T) pairs within the embedding space. Drug chemical similarity was assessed using Tanimoto similarity, and protein sequence similarity was evaluated using global Needleman–Wunsch (N–W) alignment. All analyses were performed using RDKit for chemical processing and Biopython for sequence alignment.

**Drug Interpolation Analysis** To assess how GBA-Mixup captures chemical similarity, we selected three drugs ($D_1$, $D_2$, and $D_{\mathrm{Test}}$) that share a common protein ($P_1$). The Tanimoto similarity was calculated from Extended Connectivity Fingerprints (ECFP) with a radius of 2, which captures molecular substructure information. Pairwise similarity scores (e.g., $D_1$-$D_{\mathrm{Test}}$ and $D_2$-$D_{\mathrm{Test}}$) were normalized into ratios, allowing direct comparison with Euclidean distance ratios derived from embeddings of the D–T pairs generated by the MEETA model trained with GBA-Protein Mixup.

Results showed that embedding-space distances closely mirrored the Tanimoto similarity ratios, demonstrating that GBA-Mixup encodes meaningful chemical relationships. Additionally, the GBA-Mixup model consistently achieved lower predictive errors compared to the baseline model trained without mixup, highlighting its capacity to generalize to unseen test pairs such as $D_{\mathrm{Test}}$.

**Target Interpolation Analysis** For target interpolation, we analyzed three proteins ($P_1$, $P_2$, and $P_{\text{Test}}$) associated with a common drug ($D_1$). Protein similarity was calculated using N–W global alignment with the BLOSUM62 scoring matrix and gap penalties of -10 (open) and -0.5 (extend). N–W computes an optimal full-sequence alignment, making it suitable for assessing overall homology. The resulting similarity scores (e.g., $P_1$-$P_{\text{Test}}$ and $P_2$-$P_{\text{Test}}$), measured as the number of matching characters, were normalized into ratios and compared with the Euclidean distance ratios from the embeddings of the corresponding D–T pairs.

The embedding-space distances generated by the MEETA model trained with GBA-Drug Mixup closely aligned with the N–W similarity ratios. This finding highlights that the mixup mechanism effectively encodes sequence-based relationships. Importantly, $P_{\text{Test}}$ was not included in the training set, underscoring the model's ability to generalize in zero-shot settings.

**Error Evaluation** The error measurement used in the interpolation case study is performed using absolute error on the test set, including pairs involving $D_{\text{Test}}$ and $P_{\text{Test}}$, which were unseen during training. The GBA-Mixup model consistently outperformed the baseline model, demonstrating its robustness in learning and interpolating biologically meaningful relationships.

## 3.4 Related Work

### 3.4.1 Pre-trained language models for biomolecules

Language models have revolutionized the domain of natural language processing, leveraging the power of self-supervised pre-training to complement the scarcity of labeled data. Central to their success is the development of Transformer and its attention module[10], which allows the model to learn to weigh the relative importance of tokens in a sequence, only from data. Pre-trained language models are typically trained on vast corpora using objectives such as masked token prediction, enabling them to learn contextual embeddings.

In the context of biomolecules, pre-trained language models have been applied to both molecular and protein sequences, capturing the complex patterns and relationships within these molecular entities.

**Pre-trained protein language models** Protein language models (pLMs) have emerged as powerful tools for understanding protein sequences, by treating each amino acid residue as a word token, and the whole sequence as a sentence. ProtBERT[11] and ProtT5[12], built on transformer-based architectures, are trained on large-scale protein sequence databases to derive meaningful embeddings. Other state-of-the-art models include OmegaFold[13], which incorporates structure prediction into its framework, and AlphaFold[14]-inspired embeddings that leverage 3D structural context for sequence understanding. Enhanced sequence-based models such as ESM2[15], along with its current version ESM3[16], integrate evolutionary-scale insights, advancing tasks like protein function prediction and interaction analysis.

**Pre-trained molecular language models** The adaptation of language models for molecular data has opened a new frontier for molecular representation learning. Molecular Attention Transformer (MAT)[17] and GROVER[18] stands out by using graph-based molecular embeddings, bridging the gap between graph representations and language modeling techniques. Additionally, models like ChemBERTa[19] utilize transformer architectures to process SMILES string-based representations of molecules. These models are trained on massive chemical datasets. MolFormer further refines this approach by incorporating domain-specific positional embedding strategy[20], outperforming other models on multiple benchmark tasks.

### 3.4.2 Drug-Target Affinity Prediction Models

Early DTA prediction models leveraged convolutional and recurrent neural networks to process drug and target features. These models demonstrated the feasibility of deep learning in DTA prediction but were often limited by their reliance on handcrafted feature extraction. Graph neural networks (GNNs) have gained prominence for their ability to represent molecular graphs effectively. Models like GraphDTA[21] and MGraphDTA[22] utilize GNNs to encode the spatial and relational properties of molecules and targets, offering a complementary perspective to sequence-based methods. Building on the success of transformers, several DTA models have adopted this architecture to capture sequential and contextual information from molecular and protein data through Multi-Head Attention (MHA) between protein and drug. These models were proposed to improve performance over traditional deep learning approaches.

Network-based approaches, such as CSCo-DTA[5], NeoDTI[23], and DeepDTnet[24], integrate interaction networks to model drug-target relationships. These methods combine graph-based representations with contextual embeddings, enabling comprehensive analyses of complex interaction patterns. While utilizing network knowledge is critical for performance enhancement in the biology domain, these models face a significant limitation: they are unable to predict for drugs or proteins not included in the network, making them unsuitable for unseen cases. This cold start problem highlights the dependency of these approaches on pre-existing network data, restricting their applicability to novel drug or target prediction.

### 3.4.3 Data Augmentation Through Mixup

Mixup is a data augmentation technique originating from Computer Vision domain[25], designed to enhance model generalization by linearly interpolating both input images and their corresponding labels. For a pair of samples $(x_1, y_1)$ and $(x_2, y_2)$, Mixup

generates a new sample $(\tilde{x}, \tilde{y})$ as follows:

$$\tilde{x} = \lambda x_1 + (1-\lambda)x_2, \quad \tilde{y} = \lambda y_1 + (1-\lambda)y_2$$

where $\lambda$ is a mixing ratio sampled from a Beta distribution, Beta$(\alpha, \alpha)$, with $\alpha > 0$. This method is particularly effective in regression tasks, as interpolated labels $\tilde{y}$ naturally represent intermediate relationships in continuous output spaces.

In the scenario where two DT pair nodes have an edge, the pooling embeddings and the labels are used to for linear interpolation augmenting a virtual neighbor node.

### 3.5 GBA-Mixup Model-agnosticity Experiment Setting

**Training DeepDTA[9] with GBA-Mixup** The hyperparameters for the 5-fold cross-validation experiments were modified based on those used for DeepDTA in C-Mixup[1] and summarized in the subsection (Selected Hyperparameters of MixingDTA). The experimental settings for Stage-2 are identical to those of MixingDTA. For baseline comparison experiments, the original experimental settings from the DeepDTA authors were retained.

## References


1. Yao, H., Wang, Y., Zhang, L., Zou, J. Y. & Finn, C. C-mixup: Improving generalization in regression. In Koyejo, S. *et al.* (eds.) *Advances in Neural Information Processing Systems*, vol. 35, 3361–3376 (Curran Associates, Inc., 2022).
2. Liu, T., Lin, Y., Wen, X., Jorissen, R. N. & Gilson, M. K. Bindingdb: a web-accessible database of experimentally determined protein–ligand binding affinities. *Nucleic Acids Res.* **35**, D198–D201 (2006).
3. Huang, K. *et al.* Deeppurpose: a deep learning library for drug–target interaction prediction. *Bioinformatics* **36**, 5545–5547 (2020).
4. Liu, Z. *et al.* Pdb-wide collection of binding data: current status of the pdbbind database. *Bioinformatics* **31**, 405–412 (2014).
5. Wang, J., Xiao, Y., Shang, X. & Peng, J. Predicting drug–target binding affinity with cross-scale graph contrastive learning. *Briefings Bioinforma.* **25**, bbad516 (2024).
6. HARRELL Jr., F. E., LEE, K. L. & MARK, D. B. Multivariable prognostic models: Issues in developing models, evaluating assumptions and adequacy, and measuring and reducing errors. *Stat. Medicine* **15**, 361–387 (1996).
7. Pratim Roy, P., Paul, S., Mitra, I. & Roy, K. On two novel parameters for validation of predictive qsar models. *Molecules* **14**, 1660–1701 (2009).
8. Roy, K. *et al.* Some case studies on application of "r2" metrics for judging quality of quantitative structure–activity relationship predictions: Emphasis on scaling of response data. *J. Comput. Chem.* **34**, 1071–1082 (2013).
9. Öztürk, H., Özgür, A. & Ozkirimli, E. Deepdta: deep drug–target binding affinity prediction. *Bioinformatics* **34**, i821–i829 (2018).
10. Vaswani, A. *et al.* Attention is all you need. *Adv. neural information processing systems* **30** (2017).
11. Brandes, N., Ofer, D., Peleg, Y., Rappoport, N. & Linial, M. Proteinbert: a universal deep-learning model of protein sequence and function. *Bioinformatics* **38**, 2102–2110 (2022).
12. Elnaggar, A. *et al.* Prottrans: Toward understanding the language of life through self-supervised learning. *IEEE transactions on pattern analysis machine intelligence* **44**, 7112–7127 (2021).
13. Wu, R. *et al.* High-resolution de novo structure prediction from primary sequence. *BioRxiv* 2022–07 (2022).
14. Jumper, J. *et al.* Highly accurate protein structure prediction with alphafold. *nature* **596**, 583–589 (2021).
15. Lin, Z. *et al.* Evolutionary-scale prediction of atomic-level protein structure with a language model. *Science* **379**, 1123–1130 (2023).
16. Hayes, T. *et al.* Simulating 500 million years of evolution with a language model. *Science* eads0018 (2025).
17. Maziarka, Ł. *et al.* Molecule attention transformer. *arXiv preprint arXiv:2002.08264* (2020).
18. Rong, Y. *et al.* Self-supervised graph transformer on large-scale molecular data. *Adv. neural information processing systems* **33**, 12559–12571 (2020).
19. Chithrananda, S., Grand, G. & Ramsundar, B. Chemberta: large-scale self-supervised pretraining for molecular property prediction. *arXiv preprint arXiv:2010.09885* (2020).

**20.** Su, J. *et al.* Roformer: Enhanced transformer with rotary position embedding. *Neurocomputing* **568**, 127063 (2024).

**21.** Nguyen, T. *et al.* Graphdta: predicting drug–target binding affinity with graph neural networks. *Bioinformatics* **37**, 1140–1147 (2021).

**22.** Yang, Z., Zhong, W., Zhao, L. & Chen, C. Y.-C. Mgraphdta: deep multiscale graph neural network for explainable drug–target binding affinity prediction. *Chem. science* **13**, 816–833 (2022).

**23.** Wan, F., Hong, L., Xiao, A., Jiang, T. & Zeng, J. Neodti: neural integration of neighbor information from a heterogeneous network for discovering new drug–target interactions. *Bioinformatics* **35**, 104–111 (2019).

**24.** Zeng, X. *et al.* Target identification among known drugs by deep learning from heterogeneous networks. *Chem. Sci.* **11**, 1775–1797 (2020).

**25.** Zhang, H., Cisse, M., Dauphin, Y. N. & Lopez-Paz, D. mixup: Beyond empirical risk minimization. In *International Conference on Learning Representations* (2018).
